# The JAK2/STAT3/CCND2 Axis promotes colorectal Cancer stem cell persistence and radioresistance

**DOI:** 10.1186/s13046-019-1405-7

**Published:** 2019-09-11

**Authors:** So-Yeon Park, Choong-Jae Lee, Jang-Hyun Choi, Jee-Heun Kim, Ji-Won Kim, Ji-Young Kim, Jeong-Seok Nam

**Affiliations:** 1 School of Life Sciences, Gwangju Institute of Science and Technology, Gwangju, 61005 Republic of Korea; 2 Cell Logistics Research Center, Gwangju Institute of Science and Technology, Gwangju, 61005 Republic of Korea

**Keywords:** Colorectal cancer (CRC), Radioresistance, Janus kinase 2 (JAK2), Signal transducer and activator of transcription 3 (STAT3), Cyclin D2 (CCND2), Cancer stem cells (CSCs)

## Abstract

**Background:**

Radiotherapy (RT) is a highly effective multimodal nonsurgical treatment that is essential for patients with advanced colorectal cancer (CRC). Nevertheless, cell subpopulations displaying intrinsic radioresistance survive after RT. The reactivation of their proliferation and successful colonization at local or distant sites may increase the risk of poor clinical outcomes. Recently, radioresistant cancer cells surviving RT were reported to exhibit a more aggressive phenotype than parental cells, although the underlying mechanisms remain unclear.

**Methods:**

By investigating public databases containing CRC patient data, we explored potential radioresistance-associated signaling pathways. Then, their mechanistic roles in radioresistance were investigated through multiple validation steps using patient-derived primary CRC cells, human CRC cell lines, and CRC xenografts.

**Results:**

Janus kinase (JAK)/signal transducer and activator of transcription (STAT) signaling was activated in radioresistant CRC tissues in correlation with local and distant metastases. JAK2 was preferentially overexpressed in the CRC stem cell subpopulation, which was accompanied by the phosphorylation of STAT proteins, especially STAT3. JAK2/STAT3 signaling played an essential role in promoting tumor initiation and radioresistance by limiting apoptosis and enhancing clonogenic potential. Mechanistically, the direct binding of STAT3 to the cyclin D2 (CCND2) promoter increased CCND2 transcription. CCND2 expression was required for persistent cancer stem cell (CSC) growth via the maintenance of an intact cell cycle and proliferation with low levels of DNA damage accumulation.

**Conclusion:**

Herein, we first identified JAK2/STAT3/CCND2 signaling as a resistance mechanism for the persistent growth of CSCs after RT, suggesting potential biomarkers and regimens for improving outcomes among CRC patients.

## Background

Colorectal cancer (CRC) is one of the most commonly diagnosed malignant neoplasms worldwide, with an estimated 1.8 million new cases (10.2% of the total cancer incidence) and 881,000 deaths in 2018 (9.2% of total cancer deaths) [1]. While most early-stage CRC can be cured by surgical resection, advanced CRC is difficult to completely eliminate by surgery, and multimodal treatment that includes chemotherapy and radiotherapy (RT) along with surgery is required. In particular, RT has been established as a mainstay treatment in addition to surgery for rectal cancer patients because of the close proximity of the rectum to pelvic organs, the absence of a serosa surrounding the rectum, and the technical difficulties in achieving wide surgical margins.

Overall, RT is the most important nonsurgical modality for both curative and palliative treatments of multiple types of cancer; more than half of cancer patients are treated with radiation at some point, either alone or in combination with surgery and/or chemotherapy. The primary target of the approach is nuclear DNA [2], whereby RT causes DNA damage directly via DNA ionization and indirectly via stimulation of reactive oxygen species (ROS) production. The therapeutic effects of radiation are traditionally associated with DNA double-stranded breaks, which are the most lethal form of damage to tumor cells. Nonetheless, a subpopulation of cancer cells displaying intrinsic radioresistance may survive this treatment. In addition, reactivation of proliferation in these cells and successful colonization at local or distant organs can lead to local regrowth and distant metastasis, which may increase the risk of poor clinical outcomes. In the past decade, radioresistant cancer cells in multiple types of cancer persisting after RT have been reported to display a more aggressive phenotype than their parental cells, with altered expression of genes involved in cell cycle progression, DNA damage repair, migration, and invasion [3–10]. Therefore, discovering the underlying mechanism by which radioresistant cancer cells survive and preserve their aggressive phenotype during or after RT will provide future strategies for improving the clinical outcomes of cancer patients.

The most obvious explanation for RT failure involves the cancer stem cell (CSC) population, which exhibits a self-renewing and repopulating capacity, as these cells are not readily sterilized during treatment and cause cancer recurrence [11, 12]. Various clinicopathological studies have revealed that a high frequency of CSCs in a patient’s tumor burden is correlated with a low tumor reduction rate after RT, resulting in poor clinical outcomes in multiple types of solid cancer, such as glioblastoma, head and neck squamous cell carcinoma, cervical cancer, and rectal cancer [13]. Isolation of CSCs based on putative CSC markers has revealed that CSCs possess intrinsic radioresistance through multiple biological defense mechanisms, such as increased DNA repair capacity, intracellular ROS scavenging, and cell survival pathway activation [14]. Additionally, recent experimental reports suggest that a broad range of microenvironmental stimuli can regulate CSC properties and that CSC extrinsic adaptation may contribute to radioresistance [15]. For example, CSCs are prone to shift their statuses under certain circumstances, including early dissemination to nearby tissues or the bloodstream, followed by dormancy in the circulation and reactivation of proliferation for successful colonization at distant organs. This CSC plasticity, a characteristic of these cells, has been recognized as the clinical explanation for the observed very long latent metastasis, which can occur years and even decades following apparently successful treatment of a primary tumor. Despite the importance of this process, the molecular mechanisms through which CSCs preserve their plasticity during and after therapy have not yet been fully elucidated.

In this study, we focused on identifying the signaling pathways required for radioresistance and aggressive growth after RT. By exploiting technical advances in genomics, we began our investigation through direct examination of CRC patient tissues, allowing for translational research. We examined the gene expression profiles of residual CRC tissues following RT and found the most significantly affected pathways using Ingenuity Pathway Analysis (IPA) software. We attempted to distinguish the pathways potentially related to the malignant phenotype enhanced by RT and further selected pathways also activated in CRC tissues after successful colonization at lymph nodes or distant organs. This strategy revealed activation of Janus kinase (JAK)/signal transducer and activator of transcription (STAT) signaling, and sequential in vitro and in vivo experiments showed that intrinsic JAK2 expression was elevated in CRC cells following radiation, leading to STAT phosphorylation, mainly of STAT3. By limiting apoptosis and enhancing clonogenic potential, this intrinsic activation of JAK2/STAT3 signaling in CRC cells was found to be responsible for persistent growth after RT. Interestingly, JAK2/STAT3 signaling was highly active in CSC populations, and STAT3 directly bound to the cyclin D2 (CCND2) promoter to enhance its transcription, which in turn allowed CSCs to persistently propagate after RT by stimulating the transcription of a set of genes involved in cell cycle progression, DNA synthesis, replication, and repair.

Combining these data together, we first identified JAK2/STAT3/CCND2 signaling as a resistance mechanism of CSCs for persistent growth after RT, and these findings suggest that this pathway can serve as a predictive biomarker and prime target to improve outcomes in CRC patients.

## Materials and methods

### Cell cultures and reagents

All work related to human tissues was preapproved by the Institutional Review Board (IRB) at the Gwangju Institute of Science and Technology (#20170410-BR-28-03-02) and the Lee Gil Ya Cancer and Diabetes Institute of Gachon University (GCIRB-2013-66), and this study was conducted in accordance with the Helsinki Declaration. Clinical information regarding CRC patient samples is provided in Additional file 1: Table S1 and S2. Patient-derived primary CRC cells were isolated and collected from the primary tumors of CRC patients using a Tumor Cell Isolation Kit (Milteny Biotec, Bergisch, Germany) as described in our previous report [16]. They were grown in DMEM (Welgene Inc., Daegu, Republic of Korea) supplemented with 10% FBS, 100 U/ml penicillin, and 100 U/ml streptomycin (Welgene) at 37 °C and 5% CO_2_. Human CRC cell lines, including HCT116 and LoVo, were obtained from the Korean Cell Line Bank (Seoul, Republic of Korea) and grown in RPMI 1640 (Welgene Inc., Daegu, Republic of Korea) supplemented with 5% FBS.

### Ingenuity pathway analysis

To identify possible radioresistant signaling pathways, we obtained the differentially expressed gene (DEG) lists by analyzing multiple microarray data obtained from the Gene Expression Omnibus (GEO) database. The lists of DEG cohorts used in this study are provided in Additional file 1: Table S3. By comparing irradiated tumors (*n* = 9) and nonirradiated tumors (*n* = 13) from GSE15781, we identified 3927 genes that were differentially expressed in irradiated tumors (*p*-value< 0.05, |log2 fold change|≧0.4, up: 2063 and down: 1864). The DEG list was subjected to analysis using IPA software (Ingenuity System Inc., CA, USA) to identify the canonical signaling pathways that were most significant to the DEGs. Similarly, to identify metastatic genes, we obtained a DEG list from GSE70574 and identified a total of 8884 DEGs by comparing primary CRC tissues with lymph node metastasis (*n* = 7) and nonmetastatic tissues (*n* = 9). A total of 4733 genes were upregulated, and 4151 genes were downregulated in lymph node metastatic CRC tissues (*p*-value< 0.05 and |log2 fold change|≧0.15). To compare metastatic colon cancers successfully colonizing to distant organs such as the liver and lungs (*n* = 58) with primary tumor tissues (*n* = 183), we obtained data from GSE68468 using the Georgetown Database of Cancer (G-DOC), a web platform that enables clinical research by integrating patient characteristics and clinical outcome data (https://gdoc.georgetown.edu/gdoc/). We obtained a total of 4112 DEGs, of which 2465 genes were upregulated and 1647 were downregulated in metastatic tissues (*p*-value< 0.05, and |log2 fold change|≧1.3). Metastatic signaling pathways were recognized by IPA canonical pathway analysis as described above. To find the possible upstream regulators significantly associated with the DEG list, we performed upstream regulator analysis using IPA software according to the manufacturer’s protocol.

### Real-time PCR

Total RNA was extracted from cells using RNAiso (Takara, Shiga, Japan), and RNA purity was measured using the 260/280 absorbance ratio. RNA was reverse transcribed using the PrimeScript™ 1st strand cDNA Synthesis Kit (Takara), and 600 ng of the cDNA was subjected to PCR using Power SYBR® Green PCR Master Mix (Applied Biosystems, Foster City, CA, USA). Real-time (RT)-PCR was performed using a StepOnePlus Real-Time PCR System (Applied Biosystems). The relative mRNA expression of selected genes was normalized to peptidylprolyl isomerase A (*PPIA*) and quantified using the ddCt method. The sequences of the PCR primers are listed in Additional file 1: Table S4.

### Protein isolation and Western blot assay

The cells were lysed using RIPA buffer (20 mmol/l Tris-HCl, pH 7.5, 200 mmol/l NaCl, 1% Triton X-100, 1 mmol/l dithiothreitol) containing protease inhibitor cocktail (Roche). Protein concentrations were measured with a BCA assay kit (Thermo Fisher Scientific, Waltham, MA, USA), and proteins were then separated by SDS-PAGE and transferred to polyvinylidene difluoride membranes. The membranes were then sequentially incubated with the appropriate primary antibody and horseradish peroxidase-conjugated secondary antibody. The antibodies used for the Western blot assay are listed in Additional file 1: Table S5. β-actin was used as a loading control.

### Knockdown of target genes

Small interfering RNAs (siRNAs) were purchased from Bioneer (Daejeon, Republic of Korea). siRNA transfection was performed using Lipofectamine 2000 (Invitrogen) according to the manufacturer’s protocol. Three different siRNA sequences of each target were used, and their efficiencies were measured by RT-PCR and Western blot analyses. The most effective siRNA sequence was synthesized as a short hairpin RNA (shRNA) and inserted into a lentiviral pLKO.1-puro vector (#8453, Addgene, Cambridge, MA, USA). The shRNA vector was then transfected into 293FT cells (Invitrogen) with a viral packaging mix (Sigma-Aldrich, St. Louis, MO, USA), and the viral soup was used for transfection. Puromycin (Sigma-Aldrich) was used as the selection marker. The sequences of siRNA are listed in Additional file 1: Table S6, and the sequence used for the shRNA construct is bolded.

### Statistical analysis

All statistical data were analyzed by GraphPad Prism 5.0 (GraphPad Software Inc., San Diego, CA, USA). Statistical comparisons were measured by the two-tailed Student t-test between two groups and by one-way ANOVA with Dunnett’s multiple comparison among more than three groups. The chi-squared test was used to compare the cell property distributions within different categories. For the in vivo analysis, the numbers of mice used in the experiments are indicated in the legends. Asterisks are used to indicate statistical significance; *, **, and *** indicate *p* < 0.05, *p* < 0.01, and *p* < 0.001, respectively.

More information about the methods, including the animal study, immunofluorescence, fluorescence-activated cell sorting (FACS) analysis, clonogenic assay, and chromatin immunoprecipitation assay is described in Additional file 1: Supplementary Material and Methods section.

## Results

### JAK2/STAT3 signaling is activated by radiation in colorectal cancer cells and involved in radioresistance

**Fig. 1:**
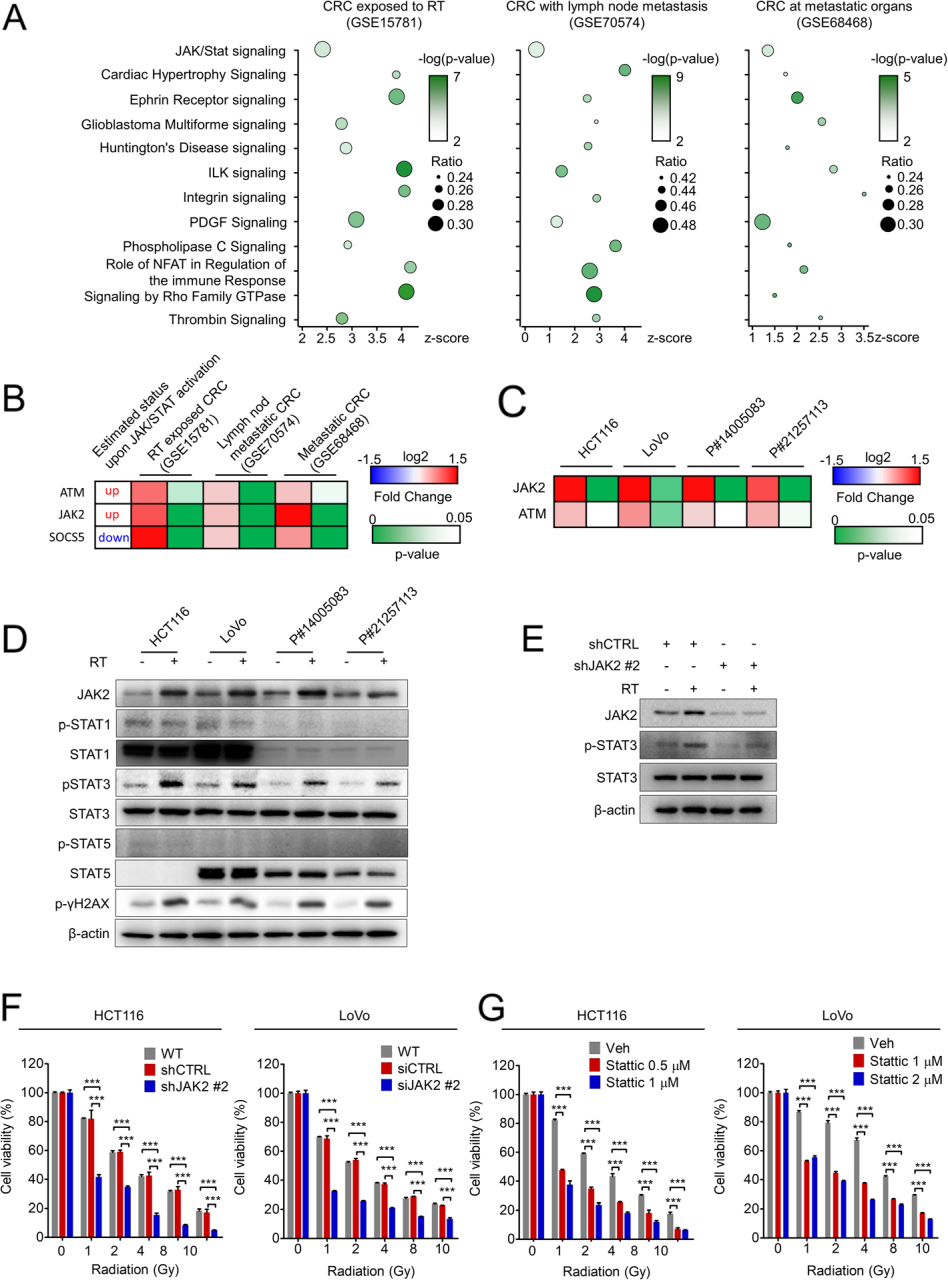
Identification of JAK2/STAT3 pathway activation as a potential radioresistance driver through CRC patient genomic analysis and multiple validations using CRC cells. **a** IPA determined that 12 signaling pathways were commonly activated in the residual CRC tissues after RT (GSE15781), in primary CRC tissues with lymph node metastasis (GSE70574), and in metastatic CRC colonizing distant organs such as the liver and lungs (GSE68468). The left Y-scale indicates enrichment z-score, the size of the round symbol indicates the ratio of contributing components, and the color indicates the *p*-value. **b** JAK/STAT signaling components that were commonly involved in all three cohorts. Two genes, *ATM* and *JAK2*, had the same tendencies between expected and experimental expression. Data are presented as the fold change compared to the control (log2) with *p*-values. **c** The mRNA expression changes in 2 potential radioresistance genes were validated in HCT116, LoVo and patient-derived primary CRC cells (P#14005083 and P#21257113) after RT (2 Gy, except for P#21257113; 4 Gy). Quantitation was conducted by real-time PCR and presented as the fold change compared to the non-RT control (log2) with *p*-values. **d** The protein expression levels of JAK2 and the p-STAT family in the above CRC cells were determined by Western blot. γH2AX was used as a positive control for radiation exposure. **e** The expression level of p-STAT3 in HCT116 cells transfected with the shCTRL vector or the shJAK2 vector with or without RT (2 Gy) was evaluated by Western blot. **f** and **g** The cell viability after RT under JAK2 knockdown or Stattic treatment conditions was assessed by the MTT assay in HCT116 (**f**) and LoVo cells (**g**). Cells were treated with various doses of radiation and then seeded in 96-well plates. After a 72-h incubation period, cell viability was quantified as the percentage of that of the non-RT control group. The bar graph represents the mean ± SD (*n* = 3). Statistical analyses were implemented using one-way ANOVA followed by Dunnett’s multiple comparison test against the control group or by Student’s t-test between two groups; *, **, and *** indicate *p* < 0.05, *p* < 0.01, and *p* < 0.001, respectively

Cancer cells displaying intrinsic radioresistance survive RT. In addition, reactivation of proliferation and successful colonization at local or distant organs by these cells may increase the risk of poor clinical outcomes. As mentioned above, radioresistant cancer cells persisting after RT have a more aggressive phenotype than their parental cells [3–9]. Despite the importance of these cells, our understanding of the molecular pathways involved in radioresistance remains fairly limited. To investigate the clinical situation, we primarily searched for signaling pathways that were most significantly altered in radioresistant CRC patient tissues. First, we determined gene expression profiles in residual CRC tissues after RT and then performed canonical signaling pathway analyses using IPA. As a result, we discovered 47 signaling pathways that were significantly upregulated after RT in radioresistant residual CRC tissues (GSE15781, Fig. 1a and Additional file 1: Table S7). Among these pathways, we focused on those possibly related to an aggressive phenotype and identified 63 pathways significantly activated in primary CRC tissues that exhibited successful colonization of lymph nodes compared with CRC tissues without lymph node metastasis (GSE70574, Fig. 1a and Additional file 1: Table S8) and 62 pathways activated in tissues from CRC that successfully colonized the liver or lung compared with primary CRC tissues (GSE68468, Fig. 1a and Additional file 1: Table S9). Upon comparing the signaling pathways identified in these three independent cohorts, IPA software predicted that 12 pathways, including JAK/STAT, renin-angiotensin, and phospholipase C signaling, might be involved in radioresistance correlating with aggressiveness. Among them, the components of the JAK/STAT pathway were most abundantly expressed in all three cohorts, with enrichment ratios of 0.313, 0.482, and 0.277 in GSE15781, GSE70574, and GSE68468, respectively. In particular, among the JAK/STAT signaling components, 3 genes, *ATM*, *JAK2*, and *SOCS5,* were universally upregulated in all three cohorts. However, according to the IPA predictions, only increased *ATM* and *JAK2* expression was linked to JAK/STAT activation, while decreased *SOCS5* transcription was predicted to activate JAK/STAT signaling (Fig. 1b, Additional file 1: Table S10-S12). Because of this inconsistency between the IPA predictions and clinical gene alterations, *SOCS5* was excluded from further validations.

Next, we hypothesized that JAK/STAT signaling, potentially related to a clinically aggressive phenotype, may be activated in CRC tissues that remain after RT. For validation, we used human colon cancer cells and patient-derived primary CRC (PD-CRC) cells, which were isolated from the primary tumors of CRC patients as previously described [16]. The transcript level of JAK2 was significantly increased after RT in all tested CRC cells, including patient-derived primary CRC cells and human CRC cell lines, while the increase in ATM transcription was statistically significant in only LoVo cells (Fig. 1c). Therefore, we focused on JAK2 in further validations. Consequently, we also confirmed that RT elevated the levels of JAK2 protein and histone H2AX phosphorylation (γ-H2AX), a marker of DNA damage (Fig. 1d), and activated its downstream target, mainly through phosphorylation of STAT3 (p-STAT3, Fig. 1d). We performed additional experiments to evaluate whether RT-induced STAT3 phosphorylation is dependent on JAK2 expression, primarily determining the silencing effects of three different small interfering RNA (siRNA) sequences targeting *JAK2* (siJAK2, Additional file 2: Figure S1A) and then constructing the short hairpin RNA (shRNA) vector using siJAK2 sequence #2, which showed the most potent silencing effect (Additional file 2: Figure S1B). Consequently, we found that JAK2 suppression significantly decreased the p-STAT3 levels in RT-exposed HCT116 cells, suggesting that JAK2 expression is required, at least in part, for RT-induced increases in p-STAT3 (Fig. 1e). Next, to confirm the functional activity of JAK2/STAT3 signaling in radioresistance, we evaluated whether the inhibition of JAK2/STAT3 signaling sensitizes CRC cells to RT. We compared the fractions of viable cells after RT with or without JAK2/STAT3 inhibition and found that HCT116 cells with JAK2 knockdown were significantly sensitized to RT, showing a more than 50% decrease in cell viability at 1 Gy of RT, whereas control cells showed only a 20% decrease at the same dose (Fig. 1f). Similarly, more than 60% of LoVo cells were viable after 1 Gy of RT, but only 30% were viable at the same dose after JAK2 knockdown (Fig. 1f and Additional file 2: Figure S1C). The radiosensitizing effect of JAK2 silencing was also observed using different siRNA sequences in HCT116 cells (Additional file 2: Figure S1D). Next, we assessed whether STAT3 inhibition has a radiosensitizing effect using Stattic, which specifically inhibits the phosphorylation, dimerization, and nuclear translocation of STAT3 with selectivity over STAT1 and STAT5 [17], and estimated the IC_50_ values of Stattic using the MTT assay to evaluate its functional effect on radioresistance with minimal cytotoxic effects. The IC_50_ values were approximately 1.045 μM and 9.164 μM in HCT116 and LoVo cells, respectively (Additional file 2: Figure S1E and S1F); in both cell types, Stattic treatment led to a selective reduction in p-STAT3 in a dose-dependent manner, though STAT1 and STAT5 remained unchanged (Additional file 2: Figure S1G and S1H). Therefore, we used these concentrations in further experiments and observed that the Stattic-mediated inhibition of STAT3 significantly reduced the fraction of viable cells after RT in a dose-dependent manner (Fig. 1g). Moreover, colonization of viable cancer cells after RT is potentially linked to the recurrence of radioresistant tumor cells. Therefore, we performed the clonogenic assay after RT under JAK2 knockdown or Stattic treatment conditions, revealing that JAK2/STAT3 inhibition reduced the clonogenic potential of HCT116 cells after RT (Additional file 2: Figure S1I and S1J). Thus, we conclude that intrinsic JAK2/STAT3 signaling is activated by RT in CRC cells and that this activation may be associated with radioresistance.

### Targeting JAK2/STAT3 sensitizes tumor cells to radiotherapy

**Fig. 2:**
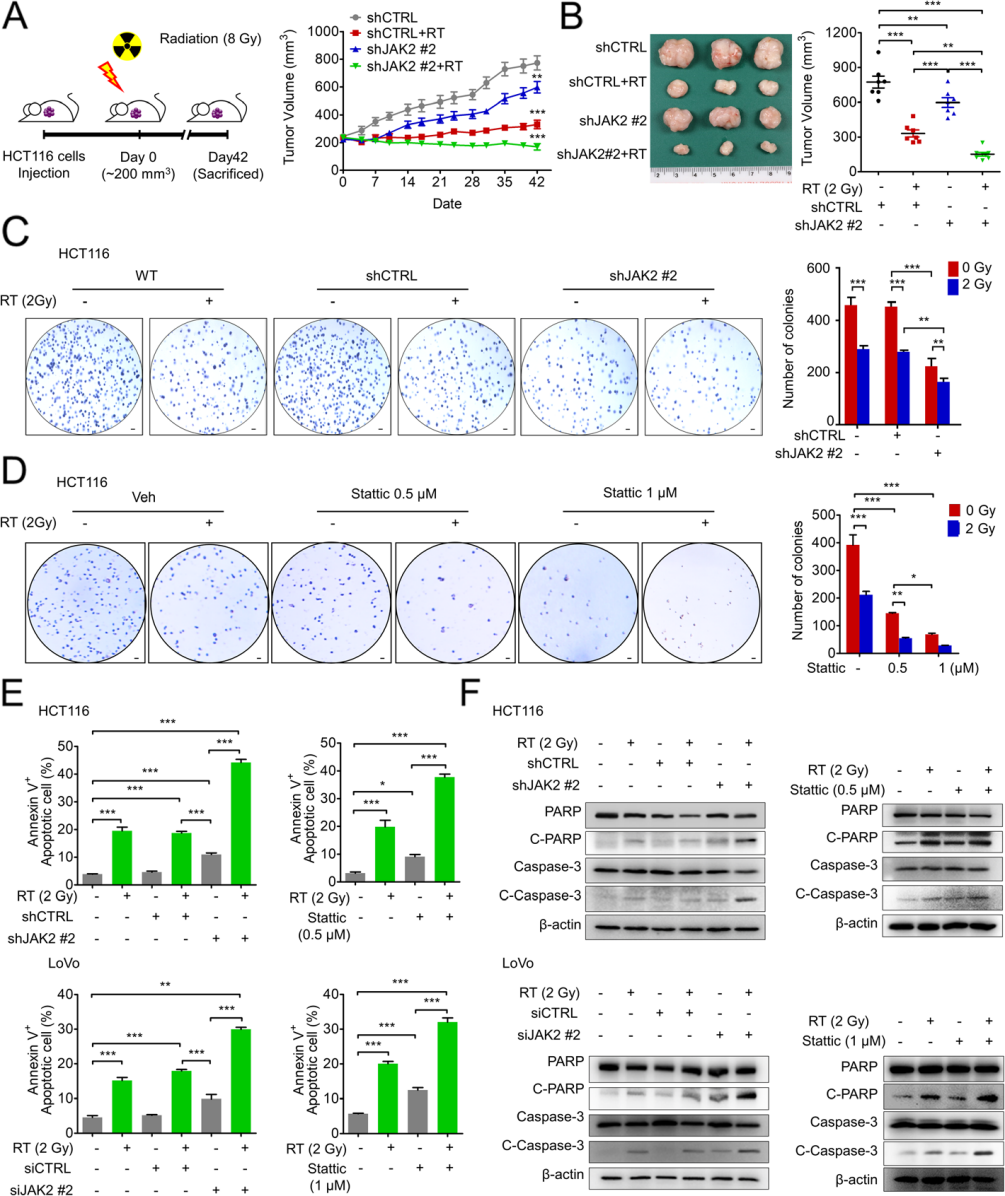
JAK2/STAT3 signaling activation is associated with a radioresistance phenotype in CRC cells. **a** HCT116 cells transfected with the shCTRL vector or shJAK2 vector were subcutaneously injected into mice. When the primary tumor volume reached approximately 200 mm^3^ (day 0), the primary tumors were irradiated to 8 Gy (shCTRL: *n* = 9; shCTRL+RT: *n* = 9; shJAK2: *n* = 9; and shJAK2 + RT: *n* = 9), and the primary tumor volume was measured twice a week until death. **b** The mice were sacrificed on day 42, and the primary tumors were isolated for subsequent experiments. The primary tumor volume on the day of sacrifice is presented. **c** and **d** The anchorage-independent growth of cells was estimated by soft agar assays. Cells with JAK2 knockdown (**c**) or Stattic treatment (**d**) were irradiated (2 Gy) and seeded on 12-well plates mixed with agar. After 2 months of incubation, the survival fraction was calculated based on the number of surviving colonies (50 μ) as a percent of that of the unirradiated control group. **e** Effect of JAK2 knockdown or Stattic treatment on radiation-induced apoptotic cells (Annexin V^+^) at 24 h after irradiation (2 Gy). **f** Radiation-induced apoptosis was evaluated by the expression level of cleaved-PARP (C-PARP) and cleaved caspase 3 (C-caspase-3) at 24 h after irradiation. Statistical analyses were implemented using one-way ANOVA followed by Dunnett’s multiple comparison test against the control group or by Student’s t-test between two groups; *, **, and *** indicate *p* < 0.05, *p* < 0.01, and *p* < 0.001, respectively

To validate the function of JAK2 in vivo, we examined whether JAK2 silencing enhances the therapeutic outcome after RT in a CRC xenograft mouse model. In the control group, the primary tumor volume remained unchanged until 3 weeks after RT, after which the tumor started to regrow (Fig. 2a). However, upon JAK2 silencing, the primary tumor volume was consistently reduced following RT. At 42 days after RT, the combination of JAK2 inhibition with RT successfully delayed tumor growth compared to that achieved with RT alone (Fig. 2a and b). Immunohistochemical analysis showed that JAK2 and p-STAT3 were elevated in the residual CRC tissue after RT; however, JAK2 knockdown diminished both the basal and radiation-induced increases in JAK2 and p-STAT3 (Additional file 3: Figure S2A and S2B). As cancer recurrence can occur due to the proliferation and colonization of viable cancer cells after RT, we evaluated whether JAK2 knockdown and STAT3 inhibition are capable of blocking the clonogenic potential of surviving cells after RT. In anchorage-independent growth (AIG) assays, JAK2 knockdown in combination with RT significantly reduced the number of colonies compared with those of both HCT116 (Fig. 2c) and LoVo cells (Additional file 3: Figure S2C) treated with RT alone. Similarly, Stattic treatment reduced the number of colonies of RT-treated CRC cells (Fig. 2d and Additional file 3: Figure S2D). FACS analysis detected an increase in Annexin V+ apoptotic cells following RT, and JAK2/STAT3 inhibition further enhanced this increase compared with that of cells treated with RT alone (Fig. 2e, Additional file 3: Figure S2E and S2F). Western blot analysis also revealed RT-induced activation of apoptotic pathway molecules. The functional forms of poly ADP-ribose polymerase and caspase-3 (cleaved-PARP and cleaved caspase-3) were elevated by RT, and JAK2 silencing or Stattic treatment further enhanced RT-induced apoptotic signaling in both HCT116 and LoVo cells compared with those of cells treated with RT alone (Fig. 2f). In accordance with the in vitro data, histopathological examination of CRC xenograft mouse model tissues revealed that although proliferating Ki67+ cells within the primary tumor were significantly reduced by RT, some cells remained; however, these cells were nearly completely eliminated by the combinatory treatment of JAK2 silencing and RT (Additional file 3: Figure S2G). Additionally, the apoptotic cell number within the primary tumor was increased by RT alone, and more importantly, apoptotic rates were further significantly enhanced by JAK2 silencing in combination with RT (Additional file 3: Figure S2H). Taken together, these data indicate that targeting JAK2/STAT3 reduces the radioresistance of CRC cells by augmenting RT-induced apoptosis and decreasing the clonogenic potential of surviving cells after RT.

### JAK2/STAT3 signaling is enriched in the CSC population among CRC cells

**Fig. 3:**
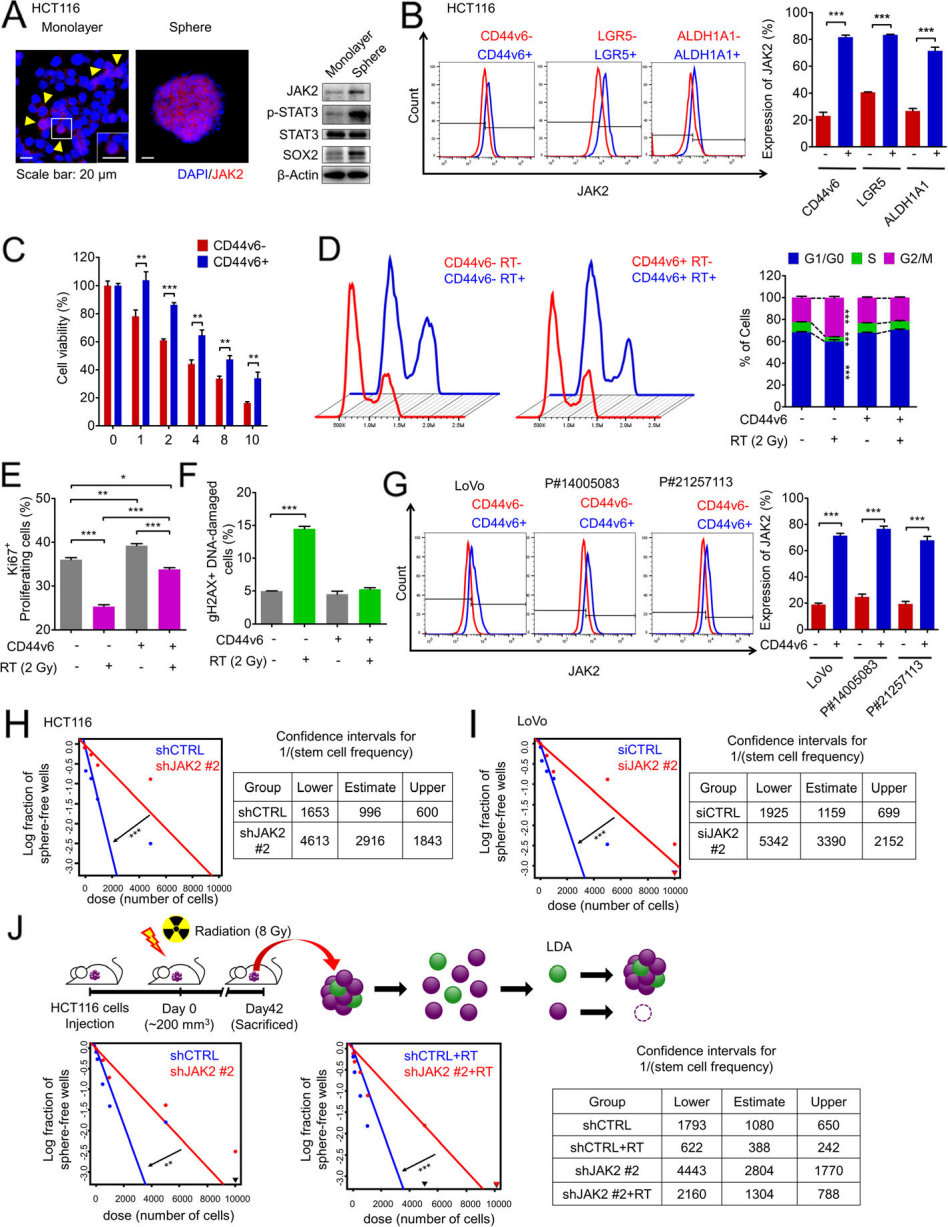
JAK2/STAT3 signaling activation is required for cancer stemness. **a** HCT116 cells were cultured under attached monolayer culture and sphere-forming conditions to enrich CSCs. Immunofluorescence assays were performed to detect JAK2 expression. Blue indicates nuclei, and red indicates JAK2. Western blot analyses visualized the increase of JAK2 protein levels as well as activation of STAT3 (p-STAT3) in CSCs. **b** FACS analysis was performed to compare the JAK2 expression between stem marker (CD44v6, LGR5 and ALDH1A1)-positive and stem marker-negative cells. **c** Cells were sorted into CD44v6+ and CD44v6- fractions. Then, their intrinsic radioresistance was determined by analyzing cell viability after RT. **d** Cell cycle analyses were performed following radiation, and cell cycle alterations were compared between the CD44v6+ and CD44v6- fractions. **e** and **f** FACS analysis revealed (**e**) Ki67+ proliferating cells or (F) γH2AX+ DNA-damaged cells in the CD44v6+ and CD44v6- fractions following radiation. **g** JAK2 expression was compared between the CD44v6+ and CD44v6- fractions in LoVo and patient-derived primary CRC cells. **h** and **i** To compare the stem cell frequencies between control cells and JAK2 knockdown cells, a limiting dilution assay was performed. **j** The primary tumors were resected from HCT116 xenograft mice as described in Fig. 2a. shCTRL- or shJAK2-transfected cancer cells were isolated from primary tumors and subjected to the LDA (12 wells/group). The bar graph represents the mean ± SD (*n* = 3). Statistical analyses were implemented using one-way ANOVA followed by Dunnett’s multiple comparison test against the control group or by Student’s t-test between two groups. The chi-squared test was used to compare the cell cycle distribution within different categories. *, **, and *** indicate *p* < 0.05, *p* < 0.01, and *p* < 0.001, respectively

Our data showed that intrinsic JAK2/STAT3 signaling was activated in RT-exposed CRC cells, contributing to radioresistance. Increasing evidence suggests that CSCs expressing putative CSC markers resist RT-induced apoptosis and therefore survive RT [11, 12, 14], and we next investigated whether CRC cells with high JAK2/STAT3 levels are related to the CSC phenotype. First, we compared JAK2 expression patterns between well-differentiated monolayer-cultured CRC cells and CSC-enriched sphere-propagated CRC cells. *JAK2* mRNA levels were elevated in sphere-cultured CRC cells compared with those in monolayer-cultured CRC cells, with increases in stemness regulators, such as *POU5F1* and *SOX2*, and decreases in differentiation markers, such as *ALPI1* and *FBP1P* (Additional file 4: Figure S3A). Although the sphere-propagated CRC cells generally exhibited high JAK2 expression, microscopic observation revealed that a subpopulation of CRC cells with higher JAK2 expression was present among most CRC cells in the monolayer (Fig. 3a and Additional file 4: Figure S3B). Consistently, Western blot analysis showed increased JAK2 and p-STAT3 expression in CSC-enriched spheres compared with that in more differentiated monolayer cells in correlation with increased expression of the stemness regulator SOX2. To investigate this phenomenon, we compared JAK2 expression levels between CSCs and non-CSCs based on putative CSC surface markers previously reported in CRC, such as CD44 variant 6 (CD44v6) [18], leucine-rich repeat-containing G-protein coupled receptor 5 (LGR5) [19], and aldehyde dehydrogenase 1 family member A1 (ALDH1A1) [20, 21], and found that JAK2 expression was highly enriched in CSC populations compared to that in their counterparts (Fig. 3b).

Recently, CD44v6 has been defined as a marker for constitutive and reprogrammed CSCs in CRC. Despite the importance of CD44v6 CSCs in CRC, their potential role in radioresistance has not yet been validated. Thus, we isolated CD44v6+ and CD44v6- populations to compare their radioresistant phenotype (Additional file 4: Figure S3C) and found a significantly higher viable cell fraction after RT for the CD44v6+ population than for the CD44v6- population (Fig. 3c). The cytotoxic effects of RT have been reported to be related to cell cycle alterations, proliferation potential loss, and DNA damage accumulation [22]; thus, we compared the biological statuses of these processes after RT. After RT, the cell cycle of the CD44v6+ population was nearly stable, though G2/M-phase arrest was enhanced, and there were fewer proliferative cells at S-phase in the CD44v6- population (Fig. 3d). Interestingly, the CD44v6+ population maintained a higher proliferative status (Ki67+) than the CD44v6- population, even after RT (Fig. 3e and Additional file 4: Figure S3D), and contained fewer apoptotic cells (Additional file 4: Figure S3E). In addition, CD44v6+ cells accumulated less DNA damage than the CD44v6- population after RT (Fig. 3f, Additional file 4: Figure S3F and S3G), suggesting that they are less sensitive for RT. Collectively, our data emphasize the importance of CD44v6+ CSCs as radioresistant CSCs, and we therefore next investigated the potential role of JAK2/STAT3 signaling in radioresistance using the CD44v6+ CSC population.

FACS analysis confirmed high protein levels of JAK2 in multiple CRC cells among the CD44v6+ CSC population (Fig. 3g). Similarly, STAT3 was preferentially activated in the CD44v6+ population of multiple CRC cells (Additional file 4: Figure S3H). Interestingly, when we knocked down JAK2 in HCT116 cells, the expression levels of a set of stemness genes, such as *POU5F1*, *SOX2*, *NANOG*, and *BMI1*, were significantly decreased (Additional file 4: Figure S3I). Therefore, we performed a limiting dilution assay (LDA) to verify whether CSC-enriched JAK2 and STAT3 activation was actually related to the stemness function, revealing that silencing JAK2 and Stattic treatment impaired the self-renewal capacity of CSCs, as the frequency of stem cells was significantly decreased by JAK2 silencing or Stattic treatment in both HCT116 and LoVo cells (Fig. 3h, i, Additional file 4: Figure S3J and S3K). Consistently, we confirmed that the sphere-forming efficiency was increased in the surviving cancer cells after radiation, while the knockdown of JAK2 reduced both the basal and RT-induced increases in sphere-forming efficiency (Additional file 4: Figure S3L), suggesting that the inhibition of JAK2 reduced the self-renewal activity of CSCs before and after radiation treatment. Next, to evaluate the therapeutic effects of JAK2 depletion on CSCs in vivo, we isolated cancer cells from the primary tumors of CRC xenograft mice (Fig. 2a) and analyzed their cancer-repopulating efficiency by the LDA. In accordance with the in vitro data, silencing JAK2 reduced the stem cell frequency within the primary tumor to one-third of that of control cells (Fig. 3j). In addition, cancer cells present in the RT-treated residual tumor burden showed a higher stem cell frequency than those in the untreated group, suggesting enrichment of the radioresistant CSC population in residual tumors following RT (Fig. 3j). Nonetheless, for JAK2-depleted cells, tumors remaining after RT harbored a lower stem cell frequency than those in the RT-alone group and even those in the untreated group (Fig. 3j). Histological analysis also confirmed the enrichment of CD44v6+ CSCs in remnant tumor tissues following RT, and JAK2 inhibition successfully eliminated these cells either alone or in combination with RT (Additional file 4: Figure S3M). Further in vitro analyses showed that targeting JAK2/STAT3 signaling by JAK2 knockdown or Stattic treatment reduced the basal proportion of CD44v6+ CSCs and, more importantly, blocked the post-RT enrichment of CD44v6+ CSCs in both HCT116 and LoVo cells (Additional file 4: Figure S3N-S3Q). Taken together, our data clearly suggest that JAK2/STAT3 signaling is highly active in CSCs and required for their self-renewal capacity and radioresistance.

### CCND2 is a direct target of JAK2/STAT3 signaling

**Fig. 4:**
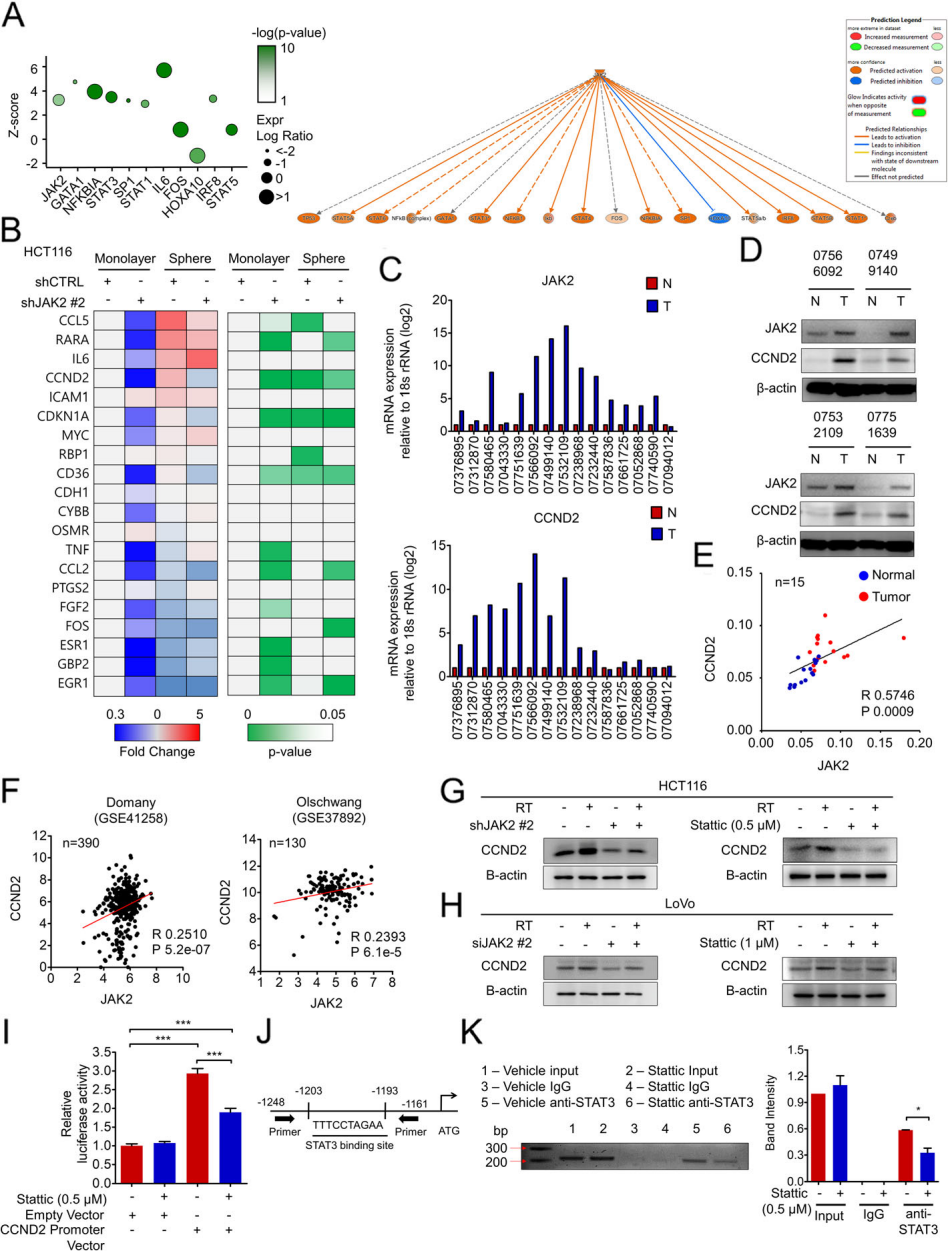
CCND2 is a downstream target of JAK2/STAT3 signaling. **a** Potential upstream regulators were analyzed in residual CRC tissues after RT (GSE 15781) using IPA software. The left Y-scale indicates enrichment z-score, the size of the round symbol indicates the ratio of contributing components, and the color indicates the *p*-value. The right panel shows the JAK2 networks interacting with other upstream regulators. **b** The potential downstream target genes of JAK2 in residual CRC tissues after RT (GSE 15781) were validated in vitro using shCTRL- or shJAK2-transfected HCT116 cells cultured in monolayer or stem cell-enriched sphere-forming conditions. **c**-**e** The expression of JAK2 and CCND2 was measured in CRC patient-derived tumor tissues and adjacent normal tissues at (**c**) mRNA and (**d**) protein levels. **e** Based on the mRNA expression level, a significant positive correlation between JAK2 and CCND2 was observed by Pearson correlation analysis. **f** In CRC patient tumors obtained from R2 database (https://hgserver1.amc.nl/cgi-bin/r2/main.cgi, GSE41258 and GSE37892), a significant positive correlation between JAK2 and CCND2 was identified by the same method above. **g** and **h** The radiation-induced expression of CCND2 was assessed by Western blot at 48 h after radiation under JAK2 knockdown or Stattic treatment in (**g**) HCT116 or (**h**) LoVo cells. **i** Transcriptional activity of STAT3 on the CCND2 promoter was confirmed by the luciferase assay with or without Stattic treatment. **j** Predicted binding sites of STAT3 on the CCND2 promoter region by the Champion ChIP Transcription Factor Search Portal (Qiagen); accordingly, primers for the ChIP assay were designed. **k** The ChIP assay was conducted in HCT116 cells with or without Stattic treatment. Immunoprecipitation was conducted using STAT3- and STAT3-bound DNA fragments amplified by PCR. PCR products were confirmed by gel electrophoresis. The bar graph represents the mean ± SD (*n* = 3). Statistical analyses were implemented using one-way ANOVA followed by Dunnett’s multiple comparison test against the control group or by Student’s t-test between two groups; *, **, and *** indicate *p* < 0.05, *p* < 0.01, and *p* < 0.001, respectively

To discover the underlying mechanism of JAK2/STAT3 in radioresistance, we analyzed the mRNA expression data among CRC patients and searched for downstream target genes. Using IPA, we identified potential upstream regulators controlling the genes differentially expressed in residual CRC tissues following RT. As expected, JAK2 was one of the activated upstream regulators, with a z-score of 3.075 and a *p*-value of 1.66E-05 (Additional file 1: Table S13). JAK2 also formed a broad network with other upstream regulators, including STAT1/3/5 complexes, the NF-κB complex, and the SP1 transcription factor. In this RT-induced signaling network, 19 genes potentially induced by JAK2 signaling were upregulated in remnant CRC tissues after RT, whereas 4 genes, possibly inhibited by the JAK2 pathway, were downregulated in the same tissues (Additional file 1: Table S14). To validate whether these genes are actually involved in JAK2/STAT3-mediated cancer cell stemness, we evaluated mRNA expression patterns in well-differentiated monolayer-cultured or CSC-enriched sphere-propagated HCT116 cells with or without JAK2 silencing. Among 19 genes potentially upregulated by the JAK2 pathway, 5 genes (*CCL5*, *CCND2*, *CDKN1A*, *RBP1*, and *CD36*) were significantly increased in CSC-enriched HCT116 cells, but only 3 genes, *CCND2*, *CDKN1A*, and *CD36*, were transcriptionally decreased after JAK2 silencing (Fig. 4b). However, both the increased *CCND2* mRNA levels in CSC-enriched spheres and the decreased levels upon JAK2 knockdown were far more significant than those in other spheres. *CCND2* encodes CCND2, a member of the D-type cyclin family that is implicated in cell cycle regulation, differentiation, and oncogenic transformation through multiple mechanisms [23]. D-type cyclins consist of three family members, cyclins D1, D2, and D3, all of which are closely related based on their mRNA sequences and protein structures. However, previous studies on cyclin D have focused on cyclin D1 because of its early discovery and widespread expression in human cancer compared with that of cyclins D2 and D3. In the present study, clinical gene expression data suggested that CCND2 is influenced by JAK2/STAT3 signaling activation in residual CRC tissues after RT. Thus, we next investigated the correlation between CCND2 and JAK2 in cancer tissues and matched normal tissues from CRC patients to analyze the expression of both CCND2 and JAK2. Both the mRNA and protein levels of JAK2 and CCND2 were enhanced in cancer tissues compared to those in normal tissues (Fig. 4c and d), and a positive correlation was observed between CCND2 and JAK2 expression in patient tissues (coefficient r value of 0.57, with statistical significance, *P* < 0.001) (Fig. 4e). Examination of CCND2 and JAK2 mRNA expression panels showed CRC tissues to be located on the right upper side of the diagram, whereas normal tissues were located on the left lower side, suggesting that overexpression of both JAK2 and CCND2 is a signature of CRC tissues compared to normal tissues. Additionally, CCND2 expression was also correlated with JAK2 expression in two independent CRC cohorts obtained from the R2 database (https://hgserver1.amc.nl/cgi-bin/r2/main.cgi), with coefficient r values of 0.25 and 0.23 in GSE41258 and GSE37892, respectively, and statistical significance at *P* < 0.001 (Fig. 4f). CCND2 protein levels were also increased following RT, similar to JAK2, and disruption of JAK2/STAT3 signaling by JAK2 silencing or Stattic treatment decreased both basal and RT-induced CCND2 expression in CRC cells (Fig. 4g and h). This phenomenon was also observed in the primary tumors of CRC xenograft mice (Additional file 5: Figure S4A). In further analyses, according to the Champion ChIP Transcription Factor Search Portal (Qiagen), the *CCND2* promoter contains one potential binding site for STAT3, from nt 4,382,004 to 4,382,013 on chromosome 12. Thus, we performed a CCND2 promoter reporter assay and observed that inhibiting STAT3 via Stattic treatment decreased the transcriptional activity of the *CCND2* promoter (Fig. 4i). Our chromatin immunoprecipitation (ChIP) assay provided the first evidence that STAT3 directly binds to the *CCND2* promoter between − 1248 and − 1161 bp and that inhibition of STAT3 phosphorylation by Stattic decreases the binding efficiency of STAT3 to the *CCND2* promoter (Fig. 4j and k). Taken together, our data clearly show that CCND2 is transcriptionally activated by JAK2/STAT3 signaling through direct binding of STAT3 to its promoter.

### CCND2 expression is required for radioresistance and cancer stemness

**Fig. 5:**
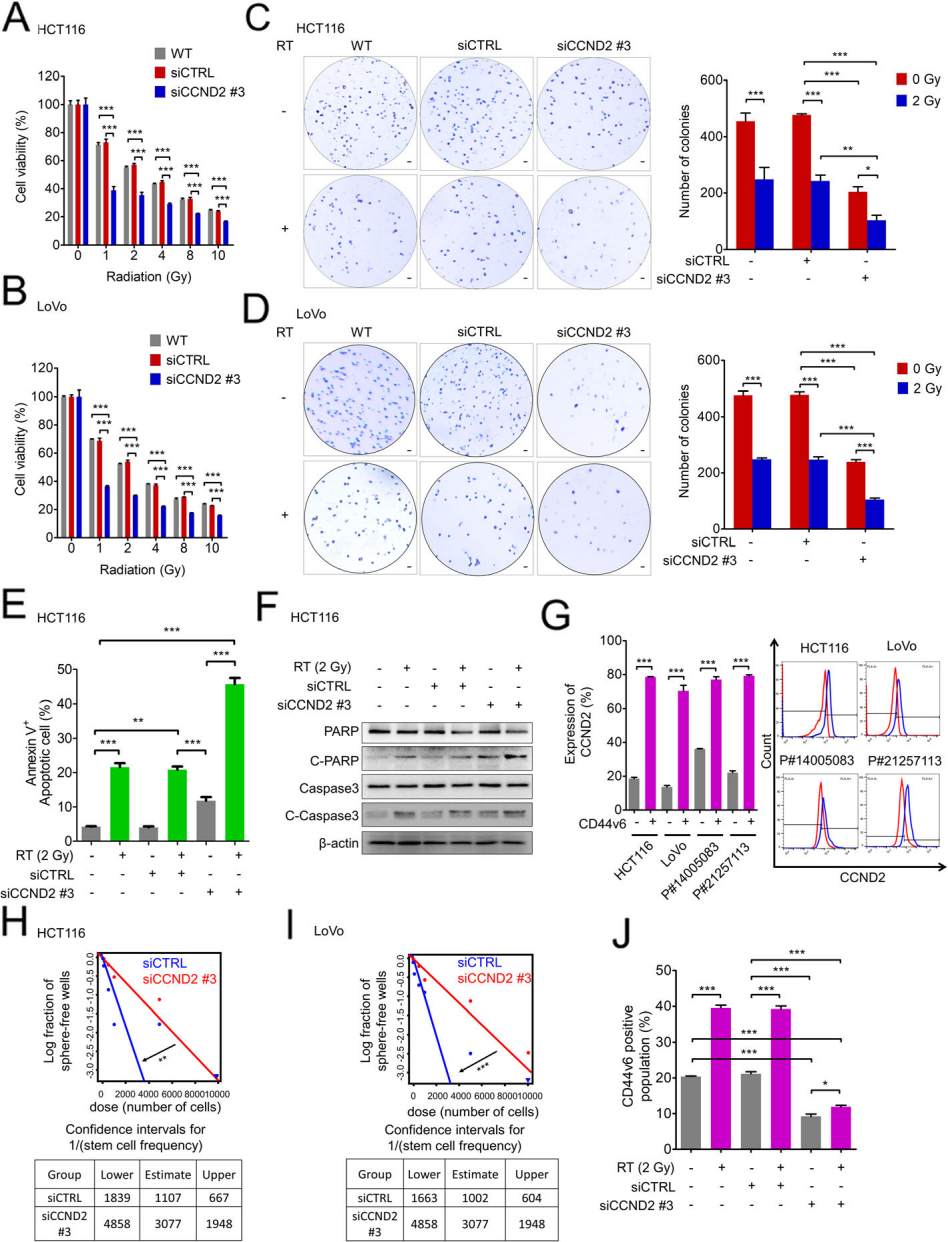
CCND2 expression is required for radioresistance and cancer stemness. **a** and **b** Cell viability after RT was assessed in CCND2 knockdown or control cells following radiation. **a** HCT116 and **b** LoVo cells were irradiated with various doses of radiation from 1 Gy to 10 Gy and cultured for 72 h. The proportion of viable cells at each radiation dose was determined by the MTT assay. **c** and **d** The anchorage-independent growth of cells after RT was estimated by soft agar assays. **c** HCT116 cells and **d** LoVo cells with or without CCND2 knockdown were irradiated (2 Gy), seeded on plates mixed with agar and incubated for 2 months. The survival fraction was calculated based on the number of surviving colonies as a percentage of that of the nonirradiated control group. **e** Effects of CCND2 knockdown on radiation-induced apoptosis (Annexin V^+^) at 24 h after radiation treatment (2 Gy). **f** Radiation-induced apoptosis was evaluated by Western blot based on cleaved-PARP and cleaved caspase-3 at 24 h after radiation treatment (2 Gy). **g** FACS analysis was performed to compare the CCND2 expression between the CD44v6+ and CD44v6- fractions in HCT116, LoVo, and patient-derived CRC cells. **h** and **i** The LDA was performed to compare the stem cell frequencies between control and CCND2 knockdown HCT116 and LoVo cells. **j** The radiation-induced enrichment of CD44v6^+^ cells was measured by FACS analysis at 24 h after radiation under CCND2 knockdown. Statistical analyses were implemented using one-way ANOVA followed by Dunnett’s multiple comparison test against the control group or by Student’s t-test between two groups; *, **, and *** indicate *p* < 0.05, *p* < 0.01, and *p* < 0.001, respectively

Because we found that CCND2 is transcriptionally activated by JAK2/STAT3 signaling in response to RT, we next evaluated whether CCND2 expression is required for radioresistance and stemness, as was JAK2/STAT3 signaling. To evaluate whether CCND2 expression is required for radioresistance, we tested the effects of knockdown using three different siRNA sequences targeting *CCND2* (siCCND2) in HCT116 cells (Additional file 6: Figure S5A). Using the most effective sequence, siCCND2 #3, we observed the radiosensitizing effect of CCND2 knockdown in HCT116 cells (Fig. 5a), in which cell viability after 1 Gy RT was reduced by more than 50% after *CCND2* knockdown; in contrast, control cells showed only a 20% decrease at the same dose (Fig. 5a). In a similar pattern, the reduction in post-RT cell viability was elevated by *CCND2* knockdown in LoVo cells (Fig. 5b and Additional file 6: Figure S5B), which was also achieved by using another sequence, siCCND2 #2, in HCT116 cells (Additional file 6: Figure S5C). An AIG assay demonstrated that silencing *CCND2* ameliorated the clonogenic potential of surviving CRC cells after RT (Fig. 5c and d). Additionally, silencing *CCND2* expression augmented RT-induced apoptosis, as determined by the detection of Annexin V+ apoptotic cells by FACS (Fig. 5e and Additional file 6: Figure S5D). Furthermore, activation of apoptotic signaling following RT was enhanced upon *CCND2* knockdown in CRC cells, resulting in increased cleavage of PARP and caspase-3 (Fig. 5f). Finally, in multiple CRC cell types, including PD-CRC, HCT116, and LoVo cells, CCND2 was preferentially expressed in CD44v6+ cells rather than in CD44v6- cells (Fig. 5g). In addition, disruption of *CCND2* expression by gene silencing reduced the self-renewal capacity of CSCs to approximately one-third of that of control cells in both HCT116 and LoVo cells (Fig. 5h and i). Moreover, CCND2 knockdown attenuated both the basal level and radiation-induced increase in sphere-forming efficiency, suggesting that CCND2 was required for the self-renewal activity of CSCs before and after radiation (Additional file 6: Figure S5E). *CCND2* knockdown reduced the CD44v6+ population, and more importantly, the post-RT enrichment of CD44v6+ CSCs was abrogated by *CCND2* knockdown (Fig. 5j and Additional file 6: Figure S5F). Collectively, our data confirm that *CCND2*, a direct target gene of JAK2/STAT3, is required for radioresistance and stemness in CRC cells.

### CD44v6+ CSC-enriched CCND2 expression allows for persistent growth after RT

**Fig. 6:**
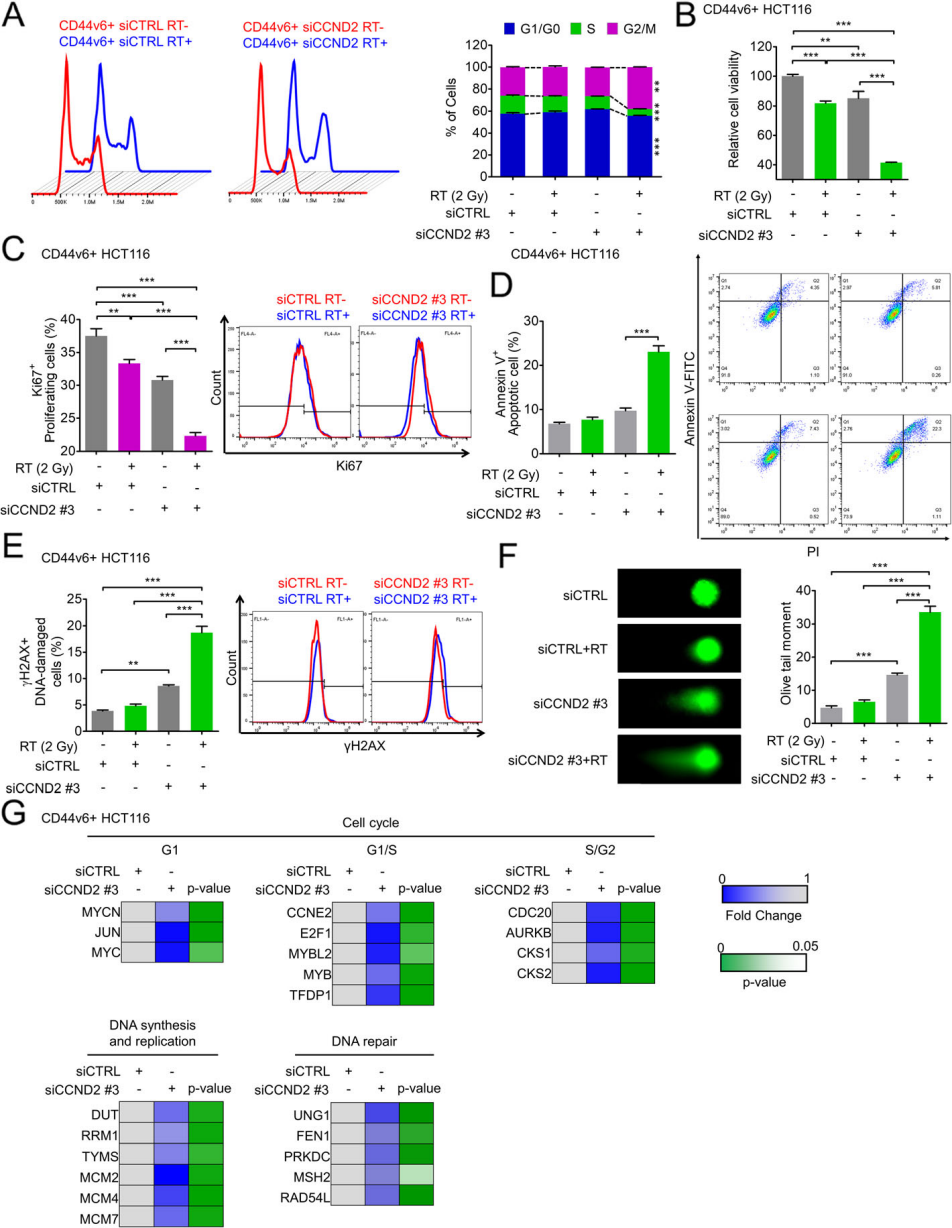
Targeting CCND2 significantly sensitizes CSCs to radiation. **a**-**g** The biological effects of CCND2 knockdown were evaluated in CD44v6+ HCT116 cells with or without RT (2 Gy). **a** Cell cycle alteration after RT was compared between siCTRL- and siCCND2-transfected CD44v6+ cells. **b** Cell viability was measured by the MTT assay at 72 h after radiation treatment. **c** and **d** FACS analysis revealed (**c**) proliferating Ki67+ cells or (**d**) Annexin V+ apoptotic cells at 24 h after radiation. **e** FACS analysis revealed γH2AX+ DNA-damaged cells 24 h after radiation. **f** Comet assay visualized the extent of DNA damage accumulation 24 h after radiation. **g** A set of genes involved in the CCND2 downstream pathway was assessed in CD44v6+ cells transfected with siCTRL or siCCND2 by real-time qPCR. The bar graph represents the mean ± SD (*n* = 3). Statistical analyses were implemented using one-way ANOVA followed by Dunnett’s multiple comparison test against the control group or by Student’s t-test between two groups. The chi-squared test was used to compare the cell cycle distribution within different categories; *, **, and *** indicate *p* < 0.05, *p* < 0.01, and *p* < 0.001, respectively

As we confirmed that CSC-enriched CCND2 expression is required for cancer stemness and radioresistance in CRC cells, we next investigated the underlying mechanism by which CCND2 is involved in radioresistance in CSCs. We isolated CD44v6+ HCT116 cells and silenced the expression of *CCND2*, which was overexpressed in CD44v6+ CSCs, and found that RT-induced cell cycle alteration could be achieved by *CCND2* knockdown in CD44v6+ CSCs (Fig. 6a), similar to the effects observed in the CD44v6- population (Fig. 3c). Additionally, targeting *CCND2* significantly decreased the post-RT viability of CD44v6+ CSCs from 80 to 40%, although it was reduced from 100% to only 80% by CCND2 knockdown (Fig. 6b). Similarly, the percentage of post-RT proliferating cells (Ki67+) among CD44v6+ CSCs was synergistically ameliorated upon silencing *CCND2* (Fig. 6c) and was correlated with increased apoptotic cell numbers (Fig. 6d). Further analysis demonstrated that *CCND2* knockdown increased the extent of DNA damage accumulation in CD44v6+ CSCs based on rH2AX staining (Fig. 6e) and the comet assay (Fig. 6f). Next, we verified that the specific subset of effector genes involved in cell cycle progression, DNA replication, synthesis, and repair were downregulated by *CCND2* knockdown in the RT-exposed CD44v6+ population (Fig. 6g). Cell cycle progression genes involved in G1 (*MYCN*, *JUN*, and *MYC*), G1/S (*CCNE2*, *E2F1*, *MYBL2*, *MYB*, and *TFDP1*), and S/G2 (*CDC20*, *AURKB*, *CKS1*, and *CKS2*) were reduced by *CCND2* silencing. Additionally, a set of genes involved in DNA synthesis and replication, including *DUT*, *RRM1*, *TYMS*, *MCM2*, *MCM4*, and *MCM7*, was abrogated by *CCND2* knockdown, as were DNA repair genes such as *UNG1*, *FEN1*, *PRKDC*, *MSH2*, and *RAD54L* (Fig. 6g). Therefore, our data provide valid evidence that CCND2 may allow CD44v6+ CSCs to grow persistently after RT by activating diverse functions such as cell cycle progression, DNA replication, and DNA repair.

Through the identification of radioresistance genes using CRC patient genomic data and validation of radioresistance mechanisms, our data provide evidence that the JAK2/STAT3/CCND2 pathway is required for persistent radioresistant CSC growth by activating CCND2 signaling. Therefore, this pathway is a potential target to improve the outcomes of patients with CRC.

## Discussion

Many studies have focused on elucidating radioresistance mechanisms to increase therapeutic efficacy among CRC patients. The results of the present study broaden the evidence related to the persistent growth of radioresistant cancer cells following RT by revealing potential therapeutic targets based on clinical genomic data with subsequent in vitro and in vivo validation. Gene expression profiling showed that the JAK/STAT signaling pathway was activated in residual CRC tissues after RT and that this activation was associated with metastasis in CRC tissues, which is the most destructive consequence resulting from aggressive cancer cells (Fig. 1a). Using multiple CRC cell types, we demonstrated that intrinsic JAK2 expression was elevated by RT, consequently leading to STAT3 activation and collectively limiting RT-induced apoptosis and enhancing the clonogenic potential of cells surviving RT (Fig. 2 and Additional file 3: Figure S2). Recently, CSCs have been identified as a strong risk factor for radioresistance and metastasis because of their plasticity. In this study, we found that JAK2/STAT3 signaling was preferentially enriched in the CSC population and required for their cancer-repopulating capacity (Fig. 3 and Additional file 4: Figure S3). As revealed by the direct binding of STAT3 to the *CCND2* promoter region, we report this gene to be a novel target of JAK2/STAT3 signaling (Fig. 4g-j). Enhanced CCND2 expression promoted persistent propagation of CSCs after RT through maintenance of an intact cell cycle and proliferation with low DNA damage accumulation (Fig. 6). Collectively, our data constitute the first identification of JAK2/STAT3/CCND2 signaling as a resistance mechanism for the persistent growth of CSCs after RT, and these findings suggest biomarkers and regimens to improve outcomes among CRC patients.

JAK family proteins are nonreceptor protein tyrosine kinases that regulate various cellular signaling pathways involving multiple cytokine receptors, such as the interleukin-6 (IL-6), erythropoietin, leptin, and interferon-γ receptors [24–26], via the phosphorylation and activation of the downstream molecule STAT. Recent data have shown that the JAK2/STAT3 pathway is preferentially activated in CD44 + CD24- breast CSCs through the excessive production of IL6 and promotes CSC growth in breast tumors [27]. Additionally, activation of the JAK/STAT pathway in glioblastoma is essential for maintenance of tumor stem cell-like phenotypic features, such as sphere formation, tumorigenicity, and expression of pluripotency-associated transcription factors [28, 29]. Conversely, disruption of constitutively activated JAK/STAT signaling results in a reduction in the CSC population and loss of tumorigenicity in vivo in a wide range of cancers, such as ovarian [30], prostate [31], glioblastoma [29], and esophageal squamous [32] cancer. In this study, we support the results of these previous reports by highlighting the importance of CCND2 as a JAK2/STAT3 signaling effector molecule that allows the persistent propagation of CSCs even under RT-induced cytotoxic damage.

CCND2, like CCND1 and CCND3, is a cyclin D-type family protein, and all three are closely related. Their kinase activities are widely known to promote oncogenic processes by enhancing cyclin-dependent kinase (CDK)-mediated signaling, which blocks the tumor suppressor retinoblastoma protein [23]. Additionally, CCNDs have noncatalytic roles, whereby interactions with chromatin-remodeling enzymes and diverse transcription factors can regulate the transcription of several gene sets involved in cell growth, DNA replication, and DNA repair [23]. In fact, CCND2 expression is increased in CRC patients with advanced disease and high TNM stages [33]. Additionally, CCND2 is frequently found in abundance in the invasive margin of CRC, acting as an independent predictor of liver metastasis in CRC patients [34]. However, to date, research on CCND2 is fairly limited compared to that on CCND1. In this study, we first determined that CCND2 expression was strongly increased in CSCs (Fig. 4b and 5g). Silencing *CCND2* abrogated the cancer-repopulating capacity of CSCs (Fig. 5h and i), resulting in a reduction in the CSC proportion among CRC cells (Fig. 5j). Moreover, silencing *CCND2* in CSCs reduced the intrinsic defensive mechanisms of CSCs, resulting in cell cycle alterations, proliferation potential loss, and DNA damage accumulation after RT (Fig. 6). Collectively, our data provide evidence that CCND2 might be involved in regulating CSC plasticity. Aberrant JAK/STAT signaling has been identified as a strong oncogenic factor involved in tumor growth, invasion, and metastasis [31, 35–43]; thus, potent JAK2 and STAT3 inhibitors have been developed [44–46] and are currently in different stages of preclinical and clinical investigations. Accordingly, our novel discovery of CCND2 as a mediator of cancer stemness under JAK2/STAT3 activation provides a rationale for developing JAK2/STAT3 inhibitors to treat intractable cancers. These findings may also facilitate the clinical use of JAK2/STAT3 inhibitors by assisting with early patient stratification and predictive biomarker identification. Moreover, we found a statistically significant positive correlation between JAK2 and CCND2 expression in multiple types of cancer, such as melanoma, breast, lung, and renal cancer (Additional file 7: Figure S6A). Therefore, our discovery of the JAK2/STAT3/CCND2 axis in CSCs will have broad applications in multiple types of cancers.

Because metastasis is a substantial life-threatening event, the development of metastasis caused by persistent cancer cells is a major concern during cancer treatment. CSCs have been reported to display therapeutic resistance as well as a high metastatic capacity. In this study, we discovered JAK2 to be a key mediator of cancer stemness, and targeting JAK2 successfully ameliorated the persistence of CSCs after RT. To investigate the possible role of JAK2 in metastasis, we directly injected JAK2-depleted CRC cells into the mouse tail vein and revealed that the number of metastatic nodules on the lungs was significantly decreased when using JAK2-depleted cells compared with that achieved using control cells (Additional file 7: Figure S6B). These data suggest that JAK2 may be involved in the regulation of direct metastasis in CRC cells. Therefore, further investigating the mechanism of JAK2 with regard to CSC metastasis will be promising for the development of future cancer therapies.

Although ROS mediate RT-induced cell death, they can impact various signaling components, ion channels, and transporters; these molecules can also modify protein kinases and the ubiquitination/proteasome system [47, 48]. In general, ROS drive the activation of mitogen-activated protein kinases (MAPKs), the most important of which are extracellular signal-regulated kinases (ERK), c-Jun N-terminal kinase (JNK), and p38 kinases. Indeed, ERK and JNK are critical for recruiting c-Fos and c-Jun to the nucleus, wherein they activate the transcription factor AP-1 [49, 50]. According to PROMO Version 3.0.2 (http://alggen.lsi.upc.es/), there are multiple potential binding sites for c-Fos, c-Jun, and AP-1 in *JAK2* promoter regions (Additional file 7: Figure S6C); thus, further investigation into the possible relationship between JAK2 and the AP-1 complex within the context of ROS will be valuable for radioresistance research.

CSCs reportedly display a radioresistant phenotype in a wide range of tumor types [51], and are known to defend themselves against DNA damage derived from chemoradiotherapy as well as high replication stress [52]. *ATM* and *ATR* are involved in DNA damage-sensing mechanisms and are known to enhance the DNA repair system of CSCs, thus promoting radioresistance [51]. Additionally, CHK1/2 and *RAD51* participate in the activation of the DNA repair process and have also been reported to be critical for the maintenance of DNA integrity in colorectal CSCs [52]. Interestingly, we found that all of these genes were transcriptionally downregulated after *CCND2* knockdown (Additional file 7: Figure S6D); therefore, our preliminary data suggest that further investigating the potential interactions between *CCND2* and these radioresistance genes may provide new insight into the mechanisms by which CSCs sense and repair damaged DNA.

Taken together, our results indicate that JAK2/STAT3/CCND2 signaling contributes to cancer stemness and radioresistance; thus, therapies that specifically target this pathway constitute a biologically driven strategy for enhancing the efficacy of radiotherapy. Further functional radiobiologic assays and analysis of JAK2/STAT3/CCND2 in a retrospective cohort of patients who were treated with RT may lead to the development of novel predictive biomarkers of radiation response in CRC.

## Conclusion

Our study demonstrated that JAK2/STAT3/CCND2 axis is a key mediator of radioresistance, leading to persistent growth of CSCs after RT in CRC. This finding suggests potential biomarkers and regimens for improving outcomes among CRC patients.

## Supplementary information

Additional file 1: Consisting of Supplementary Material and Methods, Supplementary Tables S1-S14, and Supplementary Figure legends. (ZIP 758 kb)

Additional file 2: **Figure S1.** (A) Real-time qPCR analysis of JAK2 knockdown in HCT116 cells. Based on the mRNA levels, the siRNA showing the most efficient knockdown effect was selected. (B) Real-time qPCR analysis (left) and Western blot analysis (right) of HCT116 cells transfected with JAK2-targeting shRNA (C) Real-time qPCR analysis (left) and Western blot analysis (right) of LoVo cells transfected with JAK2-targeting siRNA (D) The MTT assay was performed to assess cell viability. HCT116 cells transfected with siRNA sequence #3 were seeded in 96-well plates after being subjected to various doses of radiation. Cell viability was quantified after 72 hours of incubation. (E and F) The IC50 of Stattic was evaluated in HCT116 and LoVo cells by the MTT assay. (G and H) STAT family protein expression in HCT116 and LoVo cells under the conditions of radiation and Stattic treatment was confirmed by Western blot. (I and J) Clonogenic assays were performed using HCT116 cells. Cells were treated with radiation at various doses ranging from 1 to 10 Gy with or without (I) JAK2 silencing or (J) Stattic treatment. And then, they were seeded in 12-well plates and observed for 2 weeks. The surviving colonies were visualized by crystal violet staining. Bar graphs represent the mean ± SD (*n* = 3), and statistical analysis was performed by t-test or one-way ANOVA with Dunnett’s multiple comparison; *, **, and *** indicate *p* < 0.05, *p* < 0.01, and *p* < 0.001, respectively. (PDF 463 kb)

Additional file 3: **Figure S2.** (A and B) Immunofluorescence assays were performed to visualize the target proteins JAK2 (A) and p-STAT3 (B) in primary tumors collected from the in vivo xenograft model (*n* = 9/group). (C and D) The anchorage-independent growth of cells was estimated by soft agar assays. LoVo cells with JAK2 knockdown (C) or Stattic treatment (D) were irradiated (2 Gy), seeded in agar-layered plates and incubated for 2 months. (E andF) Effects of JAK2 knockdown or Stattic treatment on the apoptotic cell population (Annexin V+) in HCT116 (E) and LoVo cells (F) at 24 hours after radiation treatment (2 Gy). (G and H) Immunofluorescence assays were performed to visualize the target proteins Ki67 (G) and TUNEL (H) in primary tumors collected from the in vivo xenograft model (*n* = 9/group). Nuclei were stained with DAPI and matched with H&E stained images. Bar graphs represent the mean ± SD (*n* = 3), and statistical analysis was performed by t-test or one-way ANOVA with Dunnett’s multiple comparison; *, **, and *** indicate *p* < 0.05, *p* < 0.01, and *p* < 0.001, respectively. (PDF 738 kb)

Additional file 4: **Figure S3.** (A) Monolayer-cultured HCT116 cells and sphere-cultured HCT116 cells were validated by performing real-time qPCR using stem markers (POU5F1, SOX2, NANOG), differentiation markers (ALPI, FABP1) and JAK2. (B) Immunofluorescence assays were performed to compare the JAK2 expression between monolayer and sphere-cultured HCT116 cells. Blue indicates nuclei, and red indicates JAK2. (C) CD44v6+ cells and CD44v6- cells were sorted by FACS. (D) FACS analysis using Ki67 staining was performed to compare the proliferating cells between the CD44v6+ and CD44v6- populations following radiation. (E) FACS analysis using Annexin V staining was performed to compare the apoptotic cells between CD44v6+ and CD44v6- populations following radiation. (F) FACS analysis using γH2AX staining was performed to compare the radiation-induced DNA damage between the CD44v6+ and CD44v6- cell populations. (G) Comet assay was performed to compate the radiation-induced DNA damage accumulation between the CD44v6+ and CD44v6- populations following radiation. (H) Phospho-STAT3 expression was compared between the CD44v6+ and CD44v6- populations in HCT116, LoVo and patient-derived cells by FACS analysis. (I) Effects of JAK2 knockdown on mRNA levels of various CSC-related genes in HCT116 cells. (J and K) To compare the stem cell frequencies between vehicle and Stattic-treated cells, a limiting dilution assay was performed. (L) Effects of JAK2 knockdown on sphere-forming efficiency of HCT116 cells with or without radiation treatment. (M) An immunofluorescence assay was performed to visualize the target protein CD44v6 in the primary tumor collected from the in vivo xenograft model (*n* = 9/group). Nuclei were stained with DAPI and matched with H&E stained images. (N-Q) The CD44v6+ population enriched by radiation was measured by FACS analysis at 24 h after radiation with or without JAK2 silencing/Stattic treatment. Bar graphs represent the mean ± SD (*n* = 3), and statistical analysis was performed by t-test or one-way ANOVA with Dunnett’s multiple comparison; *, **, and *** indicate *p* < 0.05, *p* < 0.01, and *p* < 0.001, respectively. (PDF 1099 kb)

Additional file 5: **Figure S4.** (A) An immunofluorescence assay was performed to visualize the target proteins CCND2 in primary tumors collected from an in vivo xenograft model (*n* = 9/group). Nuclei were stained with DAPI and matched with H&E stained images. Bar graphs represent the mean ± SD (*n* = 3), and statistical analysis was performed by one-way ANOVA with Dunnett’s multiple comparison; *, **, and *** indicate *p* < 0.05, *p* < 0.01, and *p* < 0.001, respectively. (PDF 151 kb)

Additional file 6: **Figure S5.** (A) The efficiencies of three siCCND2 sequences were evaluated by real-time qPCR analysis (left) and Western blot analysis (right). (B) The selected efficient siRNA sequence was confirmed in the LoVo cell line by real-time qPCR analysis (left) and Western blot analysis (right). (C) The MTT assay was performed to assess cell viability. HCT116 cells transfected with siRNA sequence #2 were seeded in 96-well plates after being subjected to various doses of radiation. Cell viability was quantified after 72 h of incubation. (D) Effect of CCND2 knockdown on the apoptotic cell population (Annexin V+) in HCT116 cells at 24 h after radiation treatment (2 Gy). (E) Sphere-formation assay was performed to estimate the CCND2 knockdown effect on sphere-forming efficiency before and after radiation treatment in HCT116 cells. (F) The radiation-induced CD44v6+ cell population was measured by FACS analysis 24 h after radiation under CCND2 knockdown conditions. Bar graphs represent the mean ± SD (*n* = 3), and statistical analysis was performed by one-way ANOVA with Dunnett’s multiple comparison; *, **, and *** indicate *p* < 0.05, *p* < 0.01, and *p* <0.001, respectively. (PDF 265 kb)

Additional file 7: **Figure S6.** (A) A significantly positive correlation between JAK2 and CCND2 was observed in various cancers, including breast (GSE3494), lung (GSE19804), melanoma (GSE65904) and renal (GSE2712) cancer. (B) shCTRL- or shJAK2 vector-transfected HCT116 cells were injected into the tail veins of mice. At 28 days after the injection, metastatic nodules on the lungs were visualized and counted by India ink staining. The dots represent the number of metastatic nodules from each mouse, and the lines show the mean ± SEM (*n* = 9/group). (C) Predicted binding sites of AP-1, c-Jun and c-Fos on the JAK2 promoter region according to ALLGEN PROMO database version 3.0.2. (D) Effects of CCND2 knockdown on mRNA levels of various radioresistance genes. Bar graphs represent the mean ± SD (*n* = 3), and statistical analysis was performed by one-way ANOVA with Dunnett’s multiple comparison; *, **, and *** indicate *p* < 0.05, *p* < 0.01, and *p* < 0.001, respectively. (PDF 410 kb)

## Data Availability

Supplementary data are attached in additional files. Additional file 1 consists of Supplementary Material and Methods, Supplementary Tables S1-S14, and Supplementary Figure legends. Additional files 2, 3, 4, 5, 6 and 7 include Supplementary Figures S1-S6.

## Abbreviations

ALDH1A1: Aldehyde dehydrogenase 1 family member A1
CCND2: Cyclin D2
CD44v6: CD44 variant 6
ChIP: Chromatin immunoprecipitation
CRC: Colorectal cancer
CSC: Cancer stem cell
DEG: Differentially expressed gene
ERK: Extracellular signal-regulated kinase
FACS: Fluorescence-activated cell sorting
G-DOC: Georgetown Database of Cancer
GEO: Gene Expression Omnibus
IPA: Ingenuity Pathway Analysis
IRB: Institutional Review Board
JAK: Janus kinase
JNK: c-JUN N-terminal kinase
LDA: Limiting dilution assay
LGR5: Leucine-rich repeat-containing G-protein coupled receptor 5
MAPK: Mitogen-activated protein kinase
PD-CRC: Patient-derived colorectal cancer cell
PPIA: Peptidylprolyl isomerase A
ROS: Reactive oxygen species
RT: Radioresistance
RT-PCR: Real-time polymerase chain reaction
shRNA: Short hairpin RNA
siRNA: Small interfering RNA
STAT: Signal transducer and activator of transcription
