# Supplementary material for: The JAK2/STAT3/CCND2 Axis promotes colorectal Cancer stem cell persistence and radioresistance
Source: J Exp Clin Cancer Res. 2019 Sep 11;38:399. doi: 10.1186/s13046-019-1405-7 (PMC6737692; doi:10.1186/s13046-019-1405-7)
Supplement: Supplementary file 1 — Consisting of Supplementary Material and Methods, Supplementary Tables S1-S14, and Supplementary Figure legends. (ZIP 758 kb) [file 13046_2019_1405_MOESM1_ESM.zip › JECC-D-19-01127_Additional file 1_NJS.docx]

**JAK2/STAT3/CCND2 Axis Promotes Colorectal Cancer Stem Cell Persistence and Radioresistance**

So-Yeon Park^*^, Choong-Jae Lee^*^, Jang-Hyun Choi, Jee-Heun Kim, Ji-Won Kim, Ji-Young Kim, and Jeong-Seok Nam^†^

**Additional file 1 consisting of Supplementary Material and Methods, Supplementary Tables S1-S14, and Supplementary Figure legends**

**Supplementary Materials and Methods**

**Immunofluorescence and H&E staining**

Samples were fixed with 4% formalin and embedded with paraffin for immunofluorescence staining. Samples were permeabilized with 0.3 M glycine and 0.3% Triton X-100 and blocked with 2% normal swine serum (DAKO, Glostrup, Denmark). Staining was performed using primary anti-JAK2 (1:100), anti-CD44v6 (1:500), anti-Ki67 (1:500) and anti-CCND2 (LSBio LifeSpan BioSciences, Inc., Seattle, 1:100) antibodies. The secondary antibody for the unconjugated antibody was Alexa Fluor 488-conjugated rabbit IgG (Molecular Probes). All nuclei were stained with DAPI, and hematoxylin and eosin (H&E) staining was performed. Immunofluorescence images were matched with H&E-stained images. Detailed antibody information is provided in Additional file 1: Table S4.

**Flow cytometry**

Fluorescence activated cell sorting (FACS) was performed using BD Accuri^TM^ C6 (BD Biosciences). FACS data were analyzed using FlowJo software (Tree Star). Antibodies to the following proteins were used: APC-conjugated CD44 (1:200, BD Biosciences), PE-conjugated CD44v6 (3:200, BD Biosciences), APC-conjugated LGR5 (3:200, Cell Signaling Technology, Beverly, MA, USA), PE-conjugated ALDH1A1 (3:200, Cell Signaling Technologies), APC-conjugated Ki67 (1:200, Invitrogen, Carlsbad, CA, USA), Alexa Fluor 488-conjugated γH2AX (1:200, BD Biosciences), JAK2 (1:200, Cell Signaling Technologies), phospho-STAT3 (1:200, Cell Signaling Technologies), and CCND2 (1:200, Cell Signaling Technologies). The FACS gates were established by staining with an isotype antibody or secondary antibody. The secondary antibody for the unconjugated antibody was APC-conjugated goat anti-rabbit (1:500, Invitrogen). The enzymatic activity of aldehyde dehydrogenase (ALDH) was examined using the ALDEFUOR assay (StemCell Technologies, Vancouver, Canada). Cells were sorted into CD44v6^pos^ and CD44v6^neg^ populations on a FACS Aria instrument (BD Biosciences). Detailed antibody information is described in Additional file 1: Table S4.

**Cell viability assay after RT**

CRC cells were resuspended in medium, subsequently irradiated with various doses of RT, seeded in 96-well plates (1 x 10^4^ cells/well) and cultured for 72 hours. Cell viability was measured by the MTT assay (Sigma-Aldrich, Missouri, USA) according to the manufacturer’s instructions. Viable cells were determined at a wavelength of 420 nm using an Epoch microplate reader (BioTek, Winooski, VT, USA). Cell viability was calculated as a percentage of that in the nonirradiated control group.

**Apoptosis assay**

Cells were stained with Annexin V-fluorescein isothiocyanate (FITC) and propidium iodide (PI) using Apoptosis Detection Kit I (BD Biosciences, San Jose, CA, USA) and analyzed by flow cytometry. Cells were collected and washed twice with PBS. The cells were resuspended in 100 μl of binding buffer, and 5 μl of FITC Annexin V and PI were then added. The mixture was incubated at room temperature for 15 minutes in the dark. After incubation, 400 μl of binding buffer was added, and the cells were analyzed by flow cytometry.

**Single-cell gel electrophoresis (neutral comet assay)**

Cells were irradiated with a single dose of 2 Gy, mixed with 0.5 % low melting point agarose (Sigma-Aldrich), spread on CometSlide microscope slides (Trevigen, MD, USA), and subjected to lysis. After electrophoresis, the slides were stained with ethidium bromide, and comets were scored (50 cells per treatment) under a fluorescence microscope (Axio Imager 2, ZEISS, Oberkochen, Germany); the data were then analyzed using ImagePro premier 9 (Image Pro Premier 9.0, Media Cybernetics, MD, USA). The comet parameter (olive tail moment) reflects the amount of unrepaired DNA released from the cells.

**Luciferase reporter assay**

The luciferase reporter assay was performed using a vector containing the STAT3 binding promoter region of CCND2 (Genecopoeia, Maryland, USA). The mixture of vector, β-galactosidase and lipofectamine was made at the ratio of 1 μg: 1 μg: 2 μl, respectively, in 50 μl of optiMEM medium/well. Twenty-four hours after the cells were transfected with the vector, the cells were treated with Stattic. Cell lysis buffer was added after 24 hours of treatment. To measure the luciferase activity, a Renilla luciferase assay system (Promega, Madison, WI, USA) was used, and activity was measured by SpectraMax L (Molecular Devices, San Jose, CA, USA) according to the manufacturer’s recommendation. Relative luciferase activity was normalized by β-galactosidase expression.

**Chromatin immunoprecipitation (ChIP) assay**

The Ez ChIP kit (Millipore, Burlington, Massachusetts, USA) was used according to the manufacturer’s recommendation. For ChIP assays, 6 x 10^6^ cells in culture were fixed in 1% formaldehyde to crosslink DNA to bound proteins, and the reaction was quenched with the addition of 0.125 M glycine. Cells were washed with PBS and collected at 4°C in the presence of protease inhibitor cocktail (Sigma). Collected cells were resuspended in 500 μl of ChIP sonication buffer (20% SDS and protease inhibitors), followed by sonication and centrifugation of fragments (200–700 bp long) at 2000×G. Fragmented chromatin supernatant was immunoprecipitated using an anti-STAT3 antibody at 4°C overnight. Protein A agarose beads were added to the chromatin-antibody complex and centrifuged to sediment the beads. The beads were washed with cold buffer, and DNA was eluted with elution buffer. DNA in the supernatant was precipitated using a high-salt method according to the manufacturer's recommendations. The extracted DNA was purified using a kit, and 10 μl of the purified DNA was used for PCR amplification with specific primers designed around the STAT3 binding site of the CCND2 promoter. The binding site of STAT3 on the CCND2 promoter was amplified using the following primer set: F: GAGCCGGACCTAATCCCTCA and R: CGGTGCAGCGTCTAGGG.

**Anchorage independent assay**

For the anchorage-independent assay, 0.35% agar (1 mL) in RPMI 1640 media was added to the bottom layer of each well in a 12 well plate. For the top layer in each well, 1x10^3^ cells were resuspended in 1 mL of a mixture comprising 0.2% agar and RRMI 1640 media. The cells were irradiated (2 Gy), and untreated cells were used as controls. The cells were incubated at 37°C for 2 weeks, after which the colonies were stained by crystal violet, photographed and counted using ImagePro premier 9 (Image Pro Premier 9.0, Media Cybernetics, MD, USA).

**MTT assay for IC_50_ determination**

To measure the IC_50_ of Stattic, 1x10^4^ cells were seeded in 96-well plates and treated with various concentrations of Stattic. Forty-eight hours later, the cells were treated with MTT (Sigma-Aldrich) in serum-free media for 1 hour. After that, MTT was removed, and DMSO (Sigma-Aldrich) was added to each well. The cell viability was determined at a wavelength of 420 nm using an Epoch microplate reader (BioTek).

**TUNEL assay**

To detect apoptotic cells in tissue, the TUNEL assay was performed using the DeadEND^TM^ Fluorometric TUNEL system (Promega). Samples were fixed with 4% formalin and embedded in paraffin for TUNEL staining. Samples were permeabilized with Protein K Solution and equilibrated with Equilibrate buffer. Staining was performed using the rTdT enzyme and nucleotide mix according to the manufacturer’s protocol. All nuclei were stained with DAPI.

**Animal study**

All animal experiments were carried out in accordance with the Institutional Animal Care and Use Committee (IACUC) of the Gwangju Institute of Science and Technology (GIST-2017-038). To evaluate the inhibitory effects of radiation with JAK2 knockdown, we used a xenograft model. HCT116 cells that were transfected with nontargeting shRNA and JAK2 targeting shRNA (1 x 10^6^ cells/mouse mixed with Matrigel) were injected into NOD scid gamma (NSG) mice (NOD. Cg-Prkdc scid Il2rg tm1Wjl/SzJ, Jackson Laboratory). After tumor cell injection, when the tumor volume reached approximately 200 mm^3^, the mice were randomly grouped (n=9/group) and irradiated by a single dose of 2 Gy. The tumor size was measured twice a week, and the tumor volume was calculated with the following formula: volume = (longitudinal x transverse^2^)/2. For metastasis models, five-week-old male NSG mice were injected with HCT116 cells transfected with nontargeting shRNA and JAK2-targeting shRNA (1 x 10^6^ cells/mouse mixed with PBS) via their tail vein. Four weeks later, the mice were sacrificed, and the metastatic nodules on their lungs were visualized by staining with Indian ink solution (15% India ink, 85% water, 3 drops NH_4_OH/100 ml). Lungs injected with India ink (Hardy Diagnostics, Santa Maria, CA, USA) were washed in Feket's solution (300 ml of 70% EtOH, 30 ml of 37% formaldehyde, 5 ml of glacial acetic acid), and the number of metastatic nodules was counted.

**Limiting dilution assay (LDA)**

For the *in vitro* LDA, CRC cells were diluted to different degrees (1×10^4^, 5×10^3^, 1×10^3^, 5×10^2^, 1×10^2^ and 1×10^1^ cells) and seeded on poly-HEMA-coated plates (12 wells/group). After 2 weeks, the frequency of stem cells was assessed using the extreme limiting dilution assay (ELDA) webtool (http://bioinf.wehi.edu.au/ software/ elda). For the *ex vivo* LDA, cells were isolated from mouse primary tumors as described in our previous report (16). Briefly, primary tumors were minced with a sterile single-edged razor blade and sterile scissors on ice. They were then incubated with 0.1 % collagenase type IV (Sigma Aldrich) and filtered with a 100 μM filter (BD Biosciences). Contaminated red blood cells were lysed with RBC lysis buffer (Sigma Aldrich). A tumor cell isolation kit (#130-110-187, Miltenyi Biotech, Auburn, CA, USA) was used to obtain purified tumor cells, and the subsequent steps were the same as those used in the *in vitro* LDA.

**Supplementary Table S1. Clinical information regarding the CRC patient samples used for Western blot and qPCR analyses**

| **# of Patients** | **Site** | **Sex** | **Age** | **Cell metaplasia** | **Stage** | |
| --- | --- | --- | --- | --- | --- | --- |
|  |  |  |  |  | **Tumor depth** | **Lymph node metastasis** |
| 07376895 | colon | M | 78 | yes | T4a | N2a |
| 07312870 | colon | F | 73 | yes | T3 | N0 |
| 07580465 | colon | F | 78 | yes | T3 | N0 |
| 07043330 | colon | M | 72 | yes | T3 | N0 |
| 07751639 | colon | M | 60 | yes | T3 | N0 |
| 07566092 | colon | M | 81 | yes | T3 | N0 |
| 07499140 | colon | F | 80 | yes | T3 | N1a |
| 07532109 | colon | F | 72 | yes | T3 | N0 |
| 07238968 | colon | M | 61 | yes | T3 | N1a |
| 07232440 | colon | M | 77 | yes | - | - |
| 07587836 | colon | F | 66 | yes | T2 | N0 |
| 07661725 | colon | F | 54 | yes | T4a | N1a |
| 07052868 | colon | M | 58 | yes | T3 | N1a |
| 07740590 | colon | F | 77 | yes | T2 | N0 |
| 07094012 | colon | F | 62 | yes | T3 | N2a |

**Supplementary Table S2. Clinical information regarding the CRC patient samples used for establishing patient-derived primary CRC cells**

| **# of Patients** | **Sex** | **Age** | **Diagnosis** | **Surgical stage** | **Pathological diagnosis** | | | |
| --- | --- | --- | --- | --- | --- | --- | --- | --- |
|  |  |  |  |  | **K-ras** | **EGFR** | **P53** | **MSS** |
| 14005083 | M | 45 | Perforated S colon carcinoma with liver and lung metastasis | T4aN2bM1 | Wild | Mutation | Positive | MSS |
| 21257113 | M | 84 | Proximal a-colon cancer with liver metastasis | T3N1M1 | Wild | Mutation | Positive | MSS |

**Supplementary Table S3. Differentially expressed gene (DEG) lists from GSE15781, GSE70574 and GSE68468 used for Ingenuity pathway analysis (IPA)**

**(supplemented in separate excel file)**

**Supplementary Table S4. List of primer sequences used for real-time PCR**

| **Primer** | | |
| --- | --- | --- |
| **JAK2** | Forward | TCTGGGGAGTATGTTGCAGAA |
|  | Reverse | AGACATGGTTGGGTGGATACC |
| **ATM** | Forward | GAGACCGCGTGATACTGGAT |
|  | Reverse | CACGGCTTCTTTTCTCCGTT |
| **CCL2** | Forward | AGAGGCTGAGACTAACCCAGA |
|  | Reverse | GGTGACTGGGGCATTGATTG |
| **CCL5** | Forward | TCAAGACAGCACGTGGACCT |
|  | Reverse | CGGGCAATGTAGGCAAAGCA |
| **CCND2** | Forward | TCCTGGCCTCCAAACTCAAA |
|  | Reverse | AAGTCATGAGGAGTGACAGC |
| **CD36** | Forward | TGTGACTCATCAGTTCATTTCCTG |
|  | Reverse | TCCGGTCACAGCCCATTTTT |
| **CDKN1A** | Forward | ACTTTGTCACCGAGACACCA |
|  | Reverse | CAGCAGAGCAGGTGAGGTG |
| **CYBB** | Forward | AGAAGAAAGGCAAACACAACACA |
|  | Reverse | ACTTAGGTGGAATATCATAAACCCG |
| **EGR1** | Forward | CTTCAACCCTCAGGCGGACA |
|  | Reverse | CGGCCAGTATAGGTGATGGG |
| **ESR1** | Forward | CGTCGCCTCTAACCTCGG |
|  | Reverse | GGTCATGGTCATGGTCCGT |
| **FGF2** | Forward | GCTGTACTGCAAAAACGGGG |
|  | Reverse | CCTTCATAGCCAGGTAACGGT |
| **IL6** | Forward | ATGAACTCCTTCTCCACAAGCG |
|  | Reverse | ACCGAATTTGTTTGTCAATTCGT |
| **TNF** | Forward | CAGGCAGGTTCTCTTCCTCTCA |
|  | Reverse | AGGAGAAGAGGCTGAGGAACAA |
| **CDH1** | Forward | TTTGACGCCGAGAGCTACAC |
|  | Reverse | CACACCATCTGTGCCCACTT |
| **MYC** | Forward | CAAGTATACGTGGCAATGCGT |
|  | Reverse | TCAAGAGTCCCAGGGAGAGT |
| **RARA** | Forward | CTCTTGCAGCAGCCTAACCC |
|  | Reverse | AGATGCCACTCCTAGATGGG |
| **RBP1** | Forward | CCACCCGCGTAGCACC |
|  | Reverse | GATTTGCGCAAGGCCACA |
| **FOS** | Forward | CAGACTACGAGGCGTCATCC |
|  | Reverse | CGTGGGAATGAAGTTGGCAC |
| **GBP2** | Forward | TTGACAGAGCCTGGACGTTG |
|  | Reverse | AGCACTGGAAAGAAGGTTGTTC |
| **ICAM1** | Forward | ACCCCGTTGCCTAAAAAGGAG |
|  | Reverse | TGCCAGTTCCACCCGTTC |
| **OSMR** | Forward | GGAGGGAATTCCTGTGGGTC |
|  | Reverse | GGGCCGGGGCTATGAAATC |
| **PTGS2** | Forward | CCCACCCATGTCAAAACCGA |
|  | Reverse | TCCAAAATCCCTTGAAGTGGG |
| **OCT4** | Forward | GGGCTCTCCCATGCATTCAAAC |
|  | Reverse | CACCTTCCCTCCAACCAGTTGC |
| **SOX2** | Forward | TCGGCAGACTGATTCAAATA |
|  | Reverse | CCATGCAGGTTGACACCGTT |
| **NANOG** | Forward | TGGGATTTACAGGCGTGAGCCAC |
|  | Reverse | AAGCAAAGCCTCCCAATCCCAAAC |
| **ALPI** | Forward | CCAGGACATCGCCACTCAG |
|  | Reverse | TCAGTGCGGTTCCACACATA |
| **FABP1** | Forward | GGAAGGACATCAAGGGGG |
|  | Reverse | TCACCTTCCAGCTTGACGAC |
| **CCND1** | Forward | ACAAACAGATCATCCGCAAACAC |
|  | Reverse | TGTTGGGGCTCCTCAGGTTC |
| **CCND3** | Forward | GACCGACAGGCCTTGGTCAA |
|  | Reverse | AGTGCCAGTGATCCCTGCCA |
| **MYCN** | Forward | GGCGTTCCTCCTCCAACA |
|  | Reverse | CGTTCTTGGGACGCACAGT |
| **JUN** | Forward | TGTTGACAGCGGCGGAAAG |
|  | Reverse | AGCCTAAGACGCAGGAAAGG |
| **CCNE2** | Forward | TGTTGGCCACCTGTATTATCTGG |
|  | Reverse | ATCTGGAGAAATCACTTGTTCCTATTTC |
| **E2F1** | Forward | GACGGCTTGAGGGGTTGAC |
|  | Reverse | TGCTACGAAGGTCCTGACAC |
| **MYBL2** | Forward | CCCTGGTGAGGCAGTTTGG |
|  | Reverse | CTGGTCAGTGCGGTTAGGG |
| **MYB** | Forward | TGTTCCATACCCTGTAGCGTT |
|  | Reverse | TCGCTTTTCCTTCTCAGGGTC |
| **TFDP1** | Forward | AGCAGCTCTTGCCAAAAACC |
|  | Reverse | GGCGTACCAATTACCACTTGC |
| **CDC20** | Forward | GCCCACCAAGAAGGAACATC |
|  | Reverse | TTTTCCACTGAGCCGAAGGA |
| **AURKB** | Forward | CAGAAGAGCTGCACATTTGACG |
|  | Reverse | CCTTGAGCCCTAAGAGCAGATTT |
| **CKS1** | Forward | ATGTCTGAATCTGAATGGAGG |
|  | Reverse | TCATTTCTTTGGTTTCTTGGG |
| **CKS2** | Forward | GAAGAGGAGTGGAGGAGACTT |
|  | Reverse | TTTTGG AAGAGGTCGTCTAAA |
| **DUT** | Forward | GTCTCCTCGCTCGCCTTCT |
|  | Reverse | GGTGAAATGGCGGGTGTCT |
| **RRM1** | Forward | ACTAAGCACCCTGACTATGCTATCC |
|  | Reverse | ACCGCGAGGAGGATCT |
| **TYMS** | Forward | GCCTCGGTGTGCCTTTCA |
|  | Reverse | CCCGTGATGTGCGCAAT |
| **MCM2** | Forward | ACCTCACAGATTCCAGCTTCG |
|  | Reverse | TTTCATAGTATAAGTGTCTTTTT |
| **MCM4** | Forward | CGAATAGGCACAGCTCGATA |
|  | Reverse | GGCAGACACCACACACAGTT |
| **MCM7** | Forward | GGAAATATCCCTCGTAGTATCAC |
|  | Reverse | CTGAGAGTAAACCCTGTACC |
| **UNG1** | Forward | TCTCCCCGCTCCAGTTTAGA |
|  | Reverse | GCAGAGGCGGCTCAAGA |
| **FEN1** | Forward | CTGTGGACCTCATCCAGAAGCA |
|  | Reverse | CCAGCACCTCAGGTTCCAAGA |
| **PRKDC** | Forward | GCCTGCAGTCTTTGGACCC |
|  | Reverse | TCCTCCAAAACCAAAGGCTAATT |
| **MSH2** | Forward | GGCGGGAAACAGCTTAGTG |
|  | Reverse | TCTCCAACTGCAGCGTCTC |
| **RAD54L** | Forward | GAGCCCAGAGGACCTTGATA |
|  | Reverse | AACCACCTTGTCTGGACAGC |
| **IL-18** | Forward | AACAAACTATTTGTCGCAGGAA |
|  | Reverse | CTGATTCCAGGTTTTCATCATC |
| **MMP2** | Forward | AAGGATGGCAAGTACGGCTT |
|  | Reverse | AAACTTGCAGGGCTGTCCTT |
| **MMP7** | Forward | GTGGGAACAGGCTCAGGAC |
|  | Reverse | ATGACGCGGGAGTTTAACAT |

**Supplementary Table S5. List of antibodies used for Western blot, immunofluorescence and flow cytometry analyses**

| **Antibody** | | | | |  |  |
| --- | --- | --- | --- | --- | --- | --- |
| **Name** | Origin | Conjugation | Corporation | Cat# | Application |  |
| **JAK2** | Monoclonal Rabbit | Unconjugated | Cell Signaling Technology | 3230 | WB, IF, FC |  |
| **STAT3** | Monoclonal Mouse | Unconjugated | Cell Signaling Technology | 9139 | WB |  |
| **Phospho-STAT3** | Monoclonal Rabbit | Unconjugated | Cell Signaling Technology | 9145 | WB, FC |  |
| **β-ACTIN** | Monoclonal Mouse | Unconjugated | SIGMA | A5316 | WB |  |
| **PARP** | Monoclonal Rabbit | Unconjugated | Cell Signaling Technology | 9532S | WB |  |
| **CASPASE3** | Polyclonal Rabbit | Unconjugated | Cell Signaling Technology | 9662S | WB |  |
| **CD44v6** | Monoclonal Mouse | PE-conjugated | R&D SYSTEMS | FAB3660P | IF, FC |  |
| **CD44** | Monoclonal Mouse | APC-conjugated | BD Pharmingen™ | 559942 | IF, FC |  |
| **LGR5** | Monoclonal Mouse | APC-conjugated | R&D SYSTEMS | FAB8078A | FC |  |
| **ALDH1A1** | Monoclonal Rabbit | PE-conjugated | Cell Signaling Technology | 65583S | FC |  |
| **CCND2** | Monoclonal Rabbit | Unconjugated | Cell Signaling Technology | 3741 | FC |  |
| **CCND2** | Polyclonal Rabbit | Unconjugated | LSBio | LS-B13861 | WB, IF |  |
| **Ki67** | Monoclonal Mouse | APC-conjugated | Invitrogen | 17-5699-41 | FC |  |
| **Ki67** | Monoclonal Rabbit | Unconjugated | Invitrogen | MA5-14520 | IF |  |
| **γH2AX** | Polyclonal Rabbit | Unconjuagted | Abcam | ab11174 | WB |  |
| **γH2AX** | Monoclonal Mouse | Alexa Fluor™ 488 conjugated | BD Pharmingen™ | 560445 | FC |  |
| **HRP Goat Anti-Mouse Ig** | Polyclonal Goat | Peroxidase-conjugated | BD Pharmingen™ | 554002 | WB |  |
| **HRP Goat Anti-Rabbit Ig** | Polyclonal Goat | Peroxidase-conjugated | BD Pharmingen™ | 554021 | WB |  |
| **Alexa Fluor™ 488 goat anti-mouse IgG (H+L)** | | | Invitrogen | A11001 | IF |  |
| **Alexa Fluor™ 488 goat anti-rabbit IgG (H+L)** | | | Invitrogen | A11008 | IF |  |
| **Alexa Fluor™ 555 donkey anti-mouse IgG (H+L)** | | | Invitrogen | A31570 | IF |  |
| **Alexa Fluor™ 555 donkey anti-rabbit IgG (H+L)** | | | Invitrogen | A31572 | IF |  |

**Supplementary Table S6. List of siRNA sequences**

|  | Sense | Antisense |
| --- | --- | --- |
| Human JAK2 |  |  |
| #1 | GUGGUAUCACACCUGUGUA(dTdT) | UACACAGGUGUGAUACCAC(dTdT) |
| **#2** | **GUCAGUAUUAAGCAAGCAA(dTdT)** | **UUGCUUGCUUAAUACUGAC(dTdT)** |
| #3 | CAACCAUGUCUUCCAUAUA(dTdT) | UAUAUGGAAGACAUGGUUG(dTdT) |
| Human CCND2 |  |  |
| #1 | GUGUAGUUGGAUCUCUACA(dTdT) | UGUAGAGAUCCAACUACAC(dTdT) |
| #2 | GUGUGUUUAGAAGGGUUCA(dTdT) | UGAACCCUUCUAAACACAC(dTdT) |
| #3 | GUCUCUGAUCCGCAAGCAU(dTdT) | AUGCUUGCGGAUCAGAGAC(dTdT) |

***Bold sequences were used to generate the shRNA plasmid.**

**Supplementary Table S7. Signaling pathways significantly associated with the DEG list GSE15781**

| Ingenuity Canonical Pathway | -log(p-value) | Ratio | z-score |
| --- | --- | --- | --- |
| Actin Cytoskeleton Signaling | 3.99 | 0.272 | 3.051 |
| Cardiac Hypertrophy Signaling | 3.6 | 0.264 | 3.841 |
| Colorectal Cancer Metastasis Signaling | 5.67 | 0.291 | 4.837 |
| Corticotropin Releasing Hormone Signaling | 3.45 | 0.306 | 3.528 |
| CXCR4 Signaling | 4.3 | 0.297 | 3.086 |
| Dendritic Cell Maturation | 3.81 | 0.279 | 4.429 |
| Endothelin-1 Signaling | 7.9 | 0.342 | 2.394 |
| eNOS Signaling | 2.88 | 0.271 | 2.667 |
| Ephrin Receptor Signaling | 4.94 | 0.305 | 3.889 |
| Estrogen-Dependent Breast Cancer Signaling | 3.49 | 0.338 | 2.4 |
| FcγRIIB Signaling in B Lymphocytes | 2.63 | 0.34 | 2.668 |
| FLT3 Signaling in Hematopoietic Progenitor Cells | 3.13 | 0.318 | 2.502 |
| GDNF Family Ligand-Receptor Interactions | 2.81 | 0.316 | 2.4 |
| Glioblastoma Multiforme Signaling | 3.48 | 0.283 | 2.777 |
| GM-CSF Signaling | 3.08 | 0.329 | 3.273 |
| Growth Hormone Signaling | 3.91 | 0.346 | 2.502 |
| HGF Signaling | 4.19 | 0.322 | 2.744 |
| HMGB1 Signaling | 2.54 | 0.271 | 3.888 |
| Huntington's Disease Signaling | 2.82 | 0.249 | 2.846 |
| IGF-1 Signaling | 3.51 | 0.311 | 2.294 |
| IL-3 Signaling | 3.71 | 0.337 | 2.646 |
| IL-6 Signaling | 3.55 | 0.299 | 4.768 |
| IL-8 Signaling | 5.17 | 0.299 | 4.719 |
| ILK Signaling | 6.65 | 0.321 | 3.939 |
| Integrin Signaling | 4.85 | 0.288 | 3.91 |
| JAK/Stat Signaling | 2.94 | 0.313 | 2.353 |
| Leukocyte Extravasation Signaling | 12.9 | 0.386 | 3.787 |
| Mouse Embryonic Stem Cell Pluripotency | 2.83 | 0.292 | 2.335 |
| PAK Signaling | 3.95 | 0.327 | 2.335 |
| Pancreatic Adenocarcinoma Signaling | 5.08 | 0.339 | 2.556 |
| Paxillin Signaling | 5.18 | 0.345 | 2.694 |
| PDGF Signaling | 4.66 | 0.356 | 3.182 |
| Phospholipase C Signaling | 3.1 | 0.254 | 2.941 |
| PKCθ Signaling in T Lymphocytes | 2.6 | 0.273 | 4 |
| Production of Nitric Oxide and Reactive Oxygen Species in Macrophages | 3.62 | 0.275 | 4.061 |
| Renin-Angiotensin Signaling | 3.76 | 0.308 | 2.535 |
| RhoA Signaling | 3.6 | 0.303 | 2.694 |
| Role of NFAT in Regulation of the Immune Response | 4.13 | 0.286 | 4.221 |
| Role of Pattern Recognition Receptors in Recognition of Bacteria and Viruses | 4.47 | 0.314 | 3.656 |
| Signaling by Rho Family GTPases | 8.8 | 0.328 | 4.123 |
| Sphingosine-1-phosphate Signaling | 4.6 | 0.325 | 3.479 |
| Tec Kinase Signaling | 6.77 | 0.335 | 4.025 |
| Th1 Pathway | 4.65 | 0.319 | 3.888 |
| Thrombin Signaling | 5.05 | 0.296 | 2.885 |
| Thrombopoietin Signaling | 3.94 | 0.369 | 2.858 |
| TREM1 Signaling | 3.29 | 0.333 | 5 |
| VEGF Family Ligand-Receptor Interactions | 2.87 | 0.307 | 2.746 |
| -log(p-value)>2.5, z-score>2.3 |  |  |  |

**Supplementary Table S8. Signaling pathways significantly associated with the DEG list GSE70574**

| Ingenuity Canonical Pathway | -log(p-value) | Ratio | z-score |
| --- | --- | --- | --- |
| 14-3-3-mediated Signaling | 3.1 | 0.45 | 1 |
| Actin Cytoskeleton Signaling | 4.33 | 0.441 | 2.305 |
| AMPK Signaling | 6.32 | 0.477 | 0.819 |
| Androgen Signaling | 4.8 | 0.489 | 0.898 |
| cAMP-mediated signaling | 7.16 | 0.485 | 1.508 |
| Cardiac Hypertrophy Signaling | 6.38 | 0.47 | 4.04 |
| CCR3 Signaling in Eosinophils | 2.94 | 0.446 | 1.715 |
| Chemokine Signaling | 4.88 | 0.563 | 1.897 |
| Cholecystokinin/Gastrin-mediated Signaling | 3.91 | 0.495 | 2.263 |
| Corticotropin Releasing Hormone Signaling | 3.64 | 0.46 | 1.838 |
| CREB Signaling in Neurons | 4.5 | 0.448 | 2.474 |
| CXCR4 Signaling | 4.2 | 0.461 | 4.341 |
| Ephrin Receptor Signaling | 4.25 | 0.457 | 2.562 |
| ErbB Signaling | 3 | 0.469 | 1.474 |
| ERK/MAPK Signaling | 5.79 | 0.475 | 1.476 |
| ERK5 Signaling | 4.05 | 0.545 | 1.333 |
| Estrogen-Dependent Breast Cancer Signaling | 4.69 | 0.544 | 2.058 |
| fMLP Signaling in Neutrophils | 4 | 0.48 | 1.361 |
| G Beta Gamma Signaling | 2.96 | 0.456 | 3.394 |
| Glioblastoma Multiforme Signaling | 2.95 | 0.432 | 2.75 |
| GNRH Signaling | 4.49 | 0.467 | 2.646 |
| Gαi Signaling | 5.46 | 0.517 | 1.05 |
| Gαq Signaling | 5.6 | 0.491 | 3.441 |
| Huntington's Disease Signaling | 4.67 | 0.44 | 2.38 |
| IL-1 Signaling | 3.77 | 0.5 | 2.137 |
| IL-3 Signaling | 3.64 | 0.506 | 1.852 |
| IL-8 Signaling | 4.41 | 0.452 | 3.055 |
| ILK Signaling | 5.85 | 0.477 | 1.279 |
| Insulin Receptor Signaling | 4.61 | 0.482 | 1.861 |
| Integrin Signaling | 4.59 | 0.447 | 2.862 |
| JAK/Stat Signaling | 2.96 | 0.482 | 0.632 |
| LXR/RXR Activation | 4.59 | 0.496 | 1.697 |
| Mouse Embryonic Stem Cell Pluripotency | 5.78 | 0.538 | 1.457 |
| Neuroinflammation Signaling Pathway | 5.44 | 0.437 | 0.642 |
| NF-κB Signaling | 3.91 | 0.448 | 0.894 |
| NGF Signaling | 3.93 | 0.479 | 1.192 |
| NRF2-mediated Oxidative Stress Response | 3.3 | 0.43 | 0.973 |
| Opioid Signaling Pathway | 6.58 | 0.471 | 2.86 |
| P2Y Purigenic Receptor Signaling Pathway | 3.91 | 0.47 | 2.066 |
| p70S6K Signaling | 3.27 | 0.455 | 0.802 |
| PDGF Signaling | 3.04 | 0.478 | 1.372 |
| Phospholipase C Signaling | 6.11 | 0.465 | 3.386 |
| PI3K Signaling in B Lymphocytes | 2.94 | 0.446 | 1.905 |
| PKCθ Signaling in T Lymphocytes | 3.73 | 0.453 | 1.483 |
| Production of Nitric Oxide and Reactive Oxygen Species in Macrophages | 5.3 | 0.469 | 1.279 |
| Prolactin Signaling | 3.29 | 0.494 | 1.151 |
| Protein Kinase A Signaling | 7.3 | 0.442 | 0.577 |
| Rac Signaling | 3.8 | 0.479 | 2.023 |
| RANK Signaling in Osteoclasts | 4.12 | 0.5 | 1.414 |
| Relaxin Signaling | 4.52 | 0.471 | 1.581 |
| Renin-Angiotensin Signaling | 3.22 | 0.459 | 1.753 |
| RhoA Signaling | 5.97 | 0.524 | 2.324 |
| Role of NANOG in Mammalian Embryonic Stem Cell Pluripotency | 2.94 | 0.451 | 1.461 |
| Role of NFAT in Cardiac Hypertrophy | 4.33 | 0.443 | 2.502 |
| Role of NFAT in Regulation of the Immune Response | 6.61 | 0.495 | 2.588 |
| Signaling by Rho Family GTPases | 8.49 | 0.492 | 2.942 |
| Sirtuin Signaling Pathway | 2.93 | 0.401 | 1.46 |
| STAT3 Pathway | 3.91 | 0.527 | 2.082 |
| Sumoylation Pathway | 3.24 | 0.479 | 2.469 |
| Synaptic Long Term Potentiation | 3.51 | 0.467 | 1.192 |
| Thrombin Signaling | 4.77 | 0.456 | 2.778 |
| UVA-Induced MAPK Signaling | 3.27 | 0.472 | 2.414 |
| Wnt/Ca+ pathway | 5.12 | 0.587 | 0.822 |
| -log(p-value)>2.9, z-score>0.5 |  |  |  |

**Supplementary Table S9. Signaling pathways significantly associated with the DEG list GSE68468**

| Ingenuity Canonical Pathway | -log(p-value) | Ratio | z-score |
| --- | --- | --- | --- |
| Neuroinflammation Signaling Pathway | 9.19 | 0.297 | 2.556 |
| Synaptic Long Term Depression | 6.9 | 0.312 | 2.252 |
| PI3K/AKT Signaling | 6.62 | 0.341 | 1.852 |
| IL-7 Signaling Pathway | 6.45 | 0.376 | 2.121 |
| Cardiac Hypertrophy Signaling (Enhanced) | 6.32 | 0.245 | 3.104 |
| EIF2 Signaling | 6.31 | 0.289 | 3.162 |
| CREB Signaling in Neurons | 5.37 | 0.279 | 2.16 |
| VDR/RXR Activation | 5.34 | 0.372 | 1.606 |
| Adrenomedullin Signaling | 5.23 | 0.281 | 3.101 |
| B Cell Receptor Signaling | 5.16 | 0.284 | 3.355 |
| Endothelin-1 Signaling | 5 | 0.279 | 2.832 |
| Dendritic Cell Maturation | 4.58 | 0.276 | 1.857 |
| Wnt/β-catenin Signaling | 4.56 | 0.283 | 2.53 |
| NRF2-mediated Oxidative Stress Response | 4.55 | 0.272 | 1.715 |
| Calcium Signaling | 4.55 | 0.272 | 1.567 |
| cAMP-mediated Signaling | 4.43 | 0.264 | 1.753 |
| Acute Myeloid Leukemia Signaling | 4.41 | 0.32 | 2.921 |
| Ephrin Receptor Signaling | 4.39 | 0.278 | 1.982 |
| Role of NFAT in Cardiac Hypertrophy | 4.31 | 0.262 | 1.54 |
| Regulation of eIF4 and p70S6K Signaling | 4.25 | 0.28 | 2.4 |
| Production of Nitric Oxide and Reactive Oxygen Species in Macrophages | 4.07 | 0.265 | 1.511 |
| Signaling by Rho Family GTPases | 4 | 0.251 | 1.511 |
| STAT3 Pathway | 3.96 | 0.289 | 3.157 |
| Phospholipase C Signaling | 3.85 | 0.249 | 1.905 |
| TGF-β Signaling | 3.84 | 0.312 | 2.746 |
| FGF Signaling | 3.81 | 0.315 | 3.024 |
| Pancreatic Adenocarcinoma Signaling | 3.75 | 0.29 | 2.353 |
| T Cell Exhaustion Signaling Pathway | 3.75 | 0.263 | 1.897 |
| ILK Signaling | 3.73 | 0.259 | 2.714 |
| Role of NFAT in Regulation of the Immune Response | 3.68 | 0.26 | 2.188 |
| Leukocyte Extravasation Signaling | 3.55 | 0.254 | 2.534 |
| PI3K Signaling in B Lymphocytes | 3.41 | 0.275 | 2.795 |
| PDGF Signaling | 3.41 | 0.297 | 1.3 |
| Inhibition of Angiogenesis by TSP1 | 3.4 | 0.412 | 2.53 |
| p70S6K Signaling | 3.39 | 0.273 | 2.197 |
| Colorectal Cancer Metastasis Signaling | 3.36 | 0.24 | 2.177 |
| Huntington's Disease Signaling | 3.35 | 0.242 | 1.976 |
| Fc Epsilon RI Signaling | 3.32 | 0.277 | 3.212 |
| GP6 Signaling Pathway | 3.3 | 0.274 | 2.333 |
| Thrombin Signaling | 3.29 | 0.247 | 2.534 |
| Acute Phase Response Signaling | 3.26 | 0.257 | 1.808 |
| Glioblastoma Multiforme Signaling | 3.2 | 0.256 | 2.655 |
| IL-6 Signaling | 3.04 | 0.266 | 2.197 |
| iCOS-iCOSL Signaling in T Helper Cells | 3.02 | 0.272 | 2.043 |
| Integrin Signaling | 3 | 0.24 | 3.677 |
| IL-2 Signaling | 2.96 | 0.307 | 1.528 |
| Rac Signaling | 2.89 | 0.268 | 2.694 |
| Prolactin Signaling | 2.89 | 0.287 | 1.46 |
| FLT3 Signaling in Hematopoietic Progenitor Cells | 2.82 | 0.284 | 1.732 |
| Synaptic Long Term Potentiation | 2.76 | 0.264 | 1.372 |
| Small Cell Lung Cancer Signaling | 2.72 | 0.287 | 1.414 |
| Gαi Signaling | 2.71 | 0.264 | 3.413 |
| CCR3 Signaling in Eosinophils | 2.69 | 0.257 | 1.8 |
| Insulin Receptor Signaling | 2.68 | 0.253 | 2 |
| Mouse Embryonic Stem Cell Pluripotency | 2.61 | 0.265 | 1.671 |
| Telomerase Signaling | 2.6 | 0.262 | 2.117 |
| Endometrial Cancer Signaling | 2.59 | 0.293 | 1.414 |
| Cardiac Hypertrophy Signaling | 2.58 | 0.228 | 1.732 |
| Amyotrophic Lateral Sclerosis Signaling | 2.56 | 0.265 | 1.8 |
| IL-17A Signaling in Airway Cells | 2.55 | 0.288 | 2.683 |
| JAK/Stat Signaling | 2.55 | 0.277 | 1.4 |
| Relaxin Signaling | 2.52 | 0.245 | 1.606 |
| -log(p-value)>2.5, z-score>1.3 |  |  |  |

**Supplementary Table S10. Changes in JAK/STAT signaling components in GSE15781**

| Symbol | Entrez Gene Name | Gene Symbol | Expr  p-value | Expr Log Ratio | Expected |
| --- | --- | --- | --- | --- | --- |
| ATM | ATM serine/threonine kinase | ATM | 0.0377 | 0.815 | Up |
| CDKN1A | Cyclin dependent kinase inhibitor 1A | CDKN1A | 6.65E-05 | 1.733 | Down |
| CEBPB | CCAAT/enhancer binding protein beta | CEBPB | 0.013 | 1.25 | Up |
| FGFR1 | Fibroblast growth factor receptor 1 | FGFR1 | 5.27E-05 | 2.67 | Up |
| FGFR2 | Fibroblast growth factor receptor 2 | FGFR2 | 0.0494 | 1.271 | Up |
| FGFR3 | Fibroblast growth factor receptor 3 | FGFR3 | 0.000267 | -2.781 | Up |
| FOS | Fos proto-oncogene, AP-1 Transcription factor subunit | FOS | 0.0118 | 1.578 | Up |
| GRB2 | Growth factor receptor bound protein 2 | GRB2 | 0.0117 | 0.77 | Up |
| IL6 | Interleukin 6 | IL6 | 0.00196 | 3.577 | Up |
| JAK2 | Janus kinase 2 | JAK2 | 0.000237 | 0.988 | Up |
| JUN | Jun proto-oncogene, AP-1 Transcription factor subunit | JUN | 0.0279 | 1.381 | Up |
| KL | Klotho | KL | 0.00327 | 1.953 | Up |
| PIAS1 | Protein inhibitor of activated STAT 1 | PIAS1 | 0.026 | 0.484 | Down |
| PIAS3 | Protein inhibitor of activated STAT 3 | PIAS3 | 0.00237 | 0.984 | Down |
| PIK3C3 | Phosphatidylinositol 3-kinase Catalytic subunit type 3 | PIK3C3 | 0.0294 | 0.583 | Up |
| PIK3C2B | Phosphatidylinositol-4-phosphate 3-kinase catalytic subunit type 2 beta | PIK3C2B | 0.00135 | -1.046 | Up |
| PIK3CA | phosphatidylinositol-4,5-bisphosphate 3-kinase catalytic subunit alpha | PIK3CA | 0.0211 | 0.671 | Up |
| PIK3CB | phosphatidylinositol-4,5-bisphosphate 3-kinase catalytic subunit beta | PIK3CB | 0.038 | -1.182 | Up |
| PTPN11 | protein tyrosine phosphatase, non-Receptor type 11 | PTPN11 | 0.0293 | 0.782 | Up |
| RRAS | RAS-related | RRAS | 0.00657 | 1.177 | Up |
| SHC1 | SHC adaptor protein 1 | SHC1 | 0.0211 | 0.711 | Up |
| SOCS5 | Suppressor of cytokine signaling 5 | SOCS5 | 0.000201 | 1.422 | Down |
| STAT1 | Signal transducer and activator of transcription 1 | STAT1 | 0.05 | 0.693 | Up |
| STAT3 | Signal transducer and activator of transcription 3 | STAT3 | 0.0366 | 0.546 | Up |
| STAT4 | Signal transducer and activator of transcription 4 | STAT4 | 0.011 | 1.003 | Up |
| TLR9 | Toll-like receptor 9 | TLR9 | 0.0144 | 1.432 | Up |

**Supplementary Table S11. Changes in JAK/STAT signaling components in GSE70574**

| Symbol | Entrez Gene Name | Gene Symbol - | Expr  p-value | Expr Log Ratio | Expected |
| --- | --- | --- | --- | --- | --- |
| AKT3 | AKT serine/threonine kinase 3 | AKT3 | 0.000718 | 0.301 | Up |
| ATM | ATM serine/threonine kinase | ATM | 5.44E-05 | 0.243 | Up |
| BCL2L1 | BCL2 like 1 | BCL2L1 | 0.00172 | -0.24 | Up |
| CDKN1A | Cyclin dependent kinase inhibitor 1A | CDKN1A | 0.00155 | 0.418 | Down |
| FGFR1 | Fibroblast growth factor receptor 1 | FGFR1 | 0.00679 | 0.163 | Up |
| FGFR4 | Fibroblast growth factor receptor 4 | FGFR4 | 2.12E-09 | -0.472 | Up |
| FOS | Fos proto-oncogene, AP-1 Transcription factor subunit | FOS | 0.00327 | 0.484 | Up |
| GAST | gastrin | GAST | 7.44E-06 | 0.386 | Up |
| GNAQ | G protein subunit alpha q | GNAQ | 0.00783 | 0.218 | Up |
| HRAS | HRas proto-oncogene, GTPase | HRAS | 0.000941 | 0.142 | Up |
| IRS1 | Insulin receptor substrate 1 | IRS1 | 0.00351 | -0.292 | Up |
| IRS2 | Insulin receptor substrate 2 | IRS2 | 4.85E-07 | -0.585 | Up |
| JAK1 | Janus kinase 1 | JAK1 | 7.4E-08 | -0.46 | Up |
| JAK2 | Janus kinase 2 | JAK2 | 1.4E-07 | 0.314 | Up |
| JAK3 | Janus kinase 3 | JAK3 | 0.000122 | 0.232 | Up |
| JUN | Jun proto-oncogene, AP-1 transcription factor subunit | JUN | 7.23E-19 | 1.07 | Up |
| KRAS | KRAS proto-oncogene, GTPase | KRAS | 0.00818 | 0.175 | Up |
| MAPK1 | Mitogen-activated protein kinase 1 | MAPK1 | 4.49E-08 | -0.328 | Up |
| MRAS | Muscle RAS oncogene homolog | MRAS | 0.0056 | -0.283 | Up |
| MTOR | Mechanistic target of rapamycin kinase | MTOR | 3.85E-06 | -0.331 | Up |
| NFKB1 | Nuclear factor kappa B subunit 1 | NFKB1 | 0.00798 | 0.148 | Up |
| NFKB2 | Nuclear factor kappa B subunit 2 | NFKB2 | 0.00836 | -0.253 | Up |
| PIK3C3 | Phosphatidylinositol 3-kinase catalytic subunit type 3 | PIK3C3 | 0.000869 | 0.297 | Up |
| PIK3C2G | Phosphatidylinositol-4-phosphate 3-kinase catalytic subunit type 2 gamma | PIK3C2G | 0.000408 | 0.168 | Up |
| PIK3CA | Phosphatidylinositol-4,5-bisphosphate 3-kinase catalytic subunit alpha | PIK3CA | 5.05E-11 | 0.384 | Up |
| PIK3CG | Phosphatidylinositol-4,5-bisphosphate 3-kinase catalytic subunit gamma | PIK3CG | 2.88E-05 | 0.575 | Up |
| PIK3R4 | Phosphoinositide-3-kinase regulatory subunit 4 | PIK3R4 | 1.81E-05 | 0.287 | Up |
| PIK3R5 | Phosphoinositide-3-kinase regulatory subunit 5 | PIK3R5 | 8.85E-05 | -0.242 | Up |
| PTPN1 | Protein tyrosine phosphatase, non-receptor type 1 | PTPN1 | 2.11E-05 | 0.407 | Down |
| RAF1 | Raf-1 proto-oncogene, serine/threonine kinase | RAF1 | 4.4E-06 | -0.354 | Up |
| RRAS | RAS-related | RRAS | 4.06E-12 | 0.563 | Up |
| RRAS2 | RAS-related 2 | RRAS2 | 0.000218 | 0.242 | Up |
| SOCS2 | Suppressor of cytokine signaling 2 | SOCS2 | 2.63E-11 | 0.655 | Down |
| SOCS3 | Suppressor of cytokine signaling 3 | SOCS3 | 0.00093 | -0.507 | Down |
| SOCS4 | Suppressor of cytokine signaling 4 | SOCS4 | 4.46E-06 | -0.381 | Down |
| SOCS5 | Suppressor of cytokine signaling 5 | SOCS5 | 9.12E-06 | 0.254 | Down |
| SOS1 | SOS Ras/Rac guanine nucleotide exchange factor 1 | SOS1 | 3.19E-06 | -0.686 | Up |
| STAT1 | Signal transducer and activator of transcription 1 | STAT1 | 0.000172 | -0.367 | Up |
| STAT6 | Signal transducer and activator of transcription 6 | STAT6 | 1.06E-05 | -0.407 | Up |
| STAT5A | Signal transducer and activator of transcription 5A | STAT5A | 1.35E-11 | 0.867 | Up |

**Supplementary Table S12. Changes in JAK/STAT signaling components in GSE68468**

| Symbol | Entrez Gene Name | Gene Symbol | Expr  p-value | Expr Fold Change | Expected |
| --- | --- | --- | --- | --- | --- |
| AKT2 | AKT serine/threonine kinase 2 | AKT2 | 0.0105 | 1.273 | Up |
| AKT3 | AKT serine/threonine kinase 3 | AKT3 | 0.00411 | 1.223 | Up |
| ATM | ATM serine/threonine kinase | ATM | 0.0466 | 1.233 | Up |
| CEBPB | CCAAT enhancer binding protein beta | CEBPB | 0.00237 | -1.773 | Up |
| FGFR2 | Fibroblast growth factor receptor 2 | FGFR2 | 0.00156 | 1.618 | Up |
| FLT3 | Fms-related tyrosine kinase 3 | FLT3 | 1.78E-09 | -2.814 |  |
| JAK2 | Janus kinase 2 | JAK2 | 1.48E-07 | 2.754 | Up |
| JAK3 | Janus kinase 3 | JAK3 | 0.0387 | -1.238 | Up |
| KRAS | KRAS proto-oncogene, GTPase | KRAS | 0.0233 | 1.368 | Up |
| MAP2K2 | Mitogen-activated protein kinase kinase 2 | MAP2K2 | 0.004 | 1.405 | Up |
| MAPK3 | Mitogen-activated protein kinase 3 | MAPK3 | 0.00847 | 1.466 | Up |
| PIAS2 | Protein inhibitor of activated STAT 2 | PIAS2 | 0.00388 | 1.25 | Down |
| PIK3R3 | Phosphoinositide-3-kinase regulatory subunit 3 | PIK3R3 | 1.76E-05 | -1.342 | Up |
| PTPN11 | Protein tyrosine phosphatase, non-receptor type 11 | PTPN11 | 3.17E-05 | 1.349 | Up |
| RELA | RELA proto-oncogene, NF-kB subunit | RELA | 0.0177 | 1.238 | Up |
| RRAS2 | RAS-related 2 | RRAS2 | 0.00628 | 1.323 | Up |
| SHC1 | SHC adaptor protein 1 | SHC1 | 0.0182 | 1.546 | Up |
| SOCS1 | Suppressor of cytokine signaling 1 | SOCS1 | 1.27E-05 | -2.26 | Down |
| SOCS3 | Suppressor of cytokine signaling 3 | SOCS3 | 2.74E-06 | 2.154 | Down |
| SOCS5 | Suppressor of cytokine signaling 5 | SOCS5 | 6.3E-07 | 1.485 | Down |
| SOS1 | SOS RasRac guanine nucleotide exchange factor 1 | SOS1 | 0.00261 | 1.672 | Up |
| SOS2 | SOS RasRho guanine nucleotide exchange factor 2 | SOS2 | 0.00501 | 1.413 | Up |
| STAT6 | Signal transducer and activator of transcription 6 | STAT6 | 0.000644 | -1.315 | Up |
| STAT5A | Signal transducer and activator of transcription 5A | STAT5A | 0.000165 | -1.329 | Up |
| STAT5B | Signal transducer and activator of transcription 5B | STAT5B | 0.000227 | -1.354 | Up |
| TYK2 | Tyrosine kinase 2 | TYK2 | 0.0116 | 1.228 | Up |

**Supplementary Table S13. Upstream regulators in irradiated tumors (GSE15781)**

| © 2000-2017 QIAGEN. All rights reserved. | | | |  |  |
| --- | --- | --- | --- | --- | --- |
| Upstream Regulator | Expr Log Ratio | Molecule Type | Predicted Activation State | Activation z-score | p-value of Overlap |
| SFTPA1 | -0.443 | Transporter | Inhibited | -3.286 | 5.95E-08 |
| TAL1 | 0.708 | Transcription regulator | Inhibited | -3.157 | 4.71E-05 |
| SREBF1 | -0.447 | Transcription regulator | Inhibited | -3.203 | 1.17E-06 |
| HNF1B | -2.934 | Transcription regulator | Inhibited | -3.488 | 8.8E-08 |
| SMAD7 | 0.496 | Transcription regulator | Inhibited | -3.785 | 4.79E-11 |
| HNF1A | -1.471 | Transcription regulator | Inhibited | -3.923 | 9.15E-06 |
| SPDEF | -1.036 | Transcription regulator | Inhibited | -4.278 | 3.6E-11 |
| TBX2 | 0.291 | Transcription regulator | Inhibited | -5.068 | 9.7E-11 |
| MYCN | -0.344 | Transcription regulator | Inhibited | -6.081 | 4.45E-06 |
| MYC | -1.32 | Transcription regulator | Inhibited | -6.278 | 4.56E-26 |
| CDH1 | -4.016 | Other | Inhibited | -3.033 | 3.61E-05 |
| FBN1 | 2.559 | Other | Inhibited | -3.07 | 8.58E-07 |
| CR1L |  | Other | Inhibited | -4.255 | 2.32E-10 |
| EP400 | -0.181 | Other | Inhibited | -4.423 | 1.55E-08 |
| RABL6 | -0.44 | Other | Inhibited | -5.775 | 4.06E-22 |
| mir-17 |  | MicroRNA | Inhibited | -3.28 | 7.27E-05 |
| miR-29b-3p (and other miRNAs w/seed AGCACCA) |  | Mature microRNA | Inhibited | -3.278 | 1.35E-08 |
| PPARG | -1.664 | Ligand-dependent Nuclear receptor | Inhibited | -3.034 | 2.17E-10 |
| AHR | 0.561 | ligand-dependent Nuclear receptor | Inhibited | -3.431 | 1.41E-12 |
| INSR | 0.07 | Kinase | Inhibited | -4.539 | 4.64E-12 |
| PKD1 | 1.019 | Ion channel | Inhibited | -4.115 | 0.000066 |
| E2f |  | Group | Inhibited | -4.458 | 1.3E-11 |
| estrogen receptor |  | Group | Inhibited | -5.906 | 8.13E-17 |
| Alpha catenin |  | Group | Inhibited | -6.49 | 1.14E-15 |
| TLR4 | 0.452 | Transmembrane receptor | Activated | 4.604 | 5.02E-07 |
| TYROBP | 1.741 | Transmembrane receptor | Activated | 3.647 | 4.42E-05 |
| IL6R | 1.175 | Transmembrane receptor | Activated | 3.503 | 1.63E-05 |
| F3 | -0.566 | Transmembrane receptor | Activated | 3.493 | 9.37E-05 |
| EDNRA | 1.686 | Transmembrane receptor | Activated | 3.148 | 3.25E-06 |
| TP53 | -0.976 | Transcription regulator | Activated | 5.871 | 1.55E-51 |
| CDKN2A | 0.771 | Transcription regulator | Activated | 5.152 | 8.98E-19 |
| NUPR1 | 1.217 | Transcription regulator | Activated | 4.784 | 6.58E-11 |
| SRF | 1.261 | Transcription regulator | Activated | 4.781 | 5.11E-08 |
| GATA1 | -0.314 | Transcription regulator | Activated | 4.749 | 1.08E-06 |
| SMAD3 | 0.303 | Transcription regulator | Activated | 4.719 | 5.5E-06 |
| STAT4 | 1.003 | Transcription regulator | Activated | 4.366 | 1.63E-07 |
| TWIST1 | 1.358 | Transcription regulator | Activated | 4.176 | 5.9E-15 |
| SPI1 | -0.183 | Transcription regulator | Activated | 3.893 | 8.12E-06 |
| SMARCA4 | -0.561 | Transcription regulator | Activated | 3.833 | 5.73E-21 |
| IRF1 | -0.416 | Transcription regulator | Activated | 3.791 | 8.66E-06 |
| NFKBIA | 1.409 | Transcription regulator | Activated | 3.719 | 2.04E-19 |
| ERG | 0.322 | Transcription regulator | Activated | 3.683 | 1.6E-07 |
| CTNNB1 | -0.107 | Transcription regulator | Activated | 3.603 | 5.39E-13 |
| STAT3 | 0.546 | Transcription regulator | Activated | 3.571 | 2.94E-11 |
| SP1 | -1.494 | Transcription regulator | Activated | 3.53 | 9.32E-24 |
| EGR1 | 1.816 | Transcription regulator | Activated | 3.528 | 2.2E-06 |
| IRF8 | -0.693 | Transcription regulator | Activated | 3.484 | 1.03E-06 |
| TCF3 |  | Transcription regulator | Activated | 3.428 | 1.06E-08 |
| NFATC2 | 0.435 | Transcription regulator | Activated | 3.373 | 1.16E-05 |
| HTT | -0.175 | Transcription regulator | Activated | 3.235 | 1.8E-07 |
| STAT1 | 0.693 | Transcription regulator | Activated | 3.037 | 1.38E-07 |
| F2 | 0.012 | Peptidase | Activated | 5.436 | 2.01E-12 |
| BNIP3L | 1.335 | Other | Activated | 4.642 | 5.88E-10 |
| RETNLB | -2.713 | Other | Activated | 3.686 | 1.9E-07 |
| NOD2 | 0.714 | Other | Activated | 3.64 | 3.38E-05 |
| PTH | -0.276 | Other | Activated | 3.607 | 1.31E-07 |
| APP | 0.183 | Other | Activated | 3.392 | 1.65E-19 |
| S100A9 | 1.466 | Other | Activated | 3.166 | 9.79E-05 |
| CD44 | 0.56 | Other | Activated | 3.027 | 2.16E-08 |
| IKBKB | 0.045 | Kinase | Activated | 5.849 | 3.81E-14 |
| MAPK14 | 0.367 | Kinase | Activated | 4.751 | 9.63E-05 |
| CHUK | 0.005 | Kinase | Activated | 4.45 | 1.82E-12 |
| RIPK2 | 0.538 | Kinase | Activated | 4.002 | 2.51E-05 |
| IKBKG | 0.594 | Kinase | Activated | 3.938 | 7.84E-07 |
| PRKCD | -0.489 | Kinase | Activated | 3.522 | 2.63E-12 |
| MAP2K1 | 0.178 | Kinase | Activated | 3.307 | 2.67E-07 |
| MAP3K1 |  | Kinase | Activated | 3.283 | 0.000004 |
| PTK2 | -0.174 | Kinase | Activated | 3.221 | 8.73E-08 |
| MKNK1 | 0.691 | Kinase | Activated | 3.087 | 6.75E-05 |
| JAK2 | 0.988 | Kinase | Activated | 3.075 | 1.66E-05 |
| IPMK | 0.275 | Kinase | Activated | 3.048 | 1.49E-05 |
| TGFB1 | 1.316 | Growth factor | Activated | 7.131 | 3.89E-64 |
| TGFB3 | 3.191 | Growth factor | Activated | 4.599 | 2.98E-12 |
| AGT | -1.513 | Growth factor | Activated | 3.626 | 5.45E-16 |
| LEP | 0.153 | Growth factor | Activated | 3.533 | 2.41E-06 |
| Interferon alpha |  | Group | Activated | 6.127 | 6.58E-06 |
| IL1 |  | Group | Activated | 4.953 | 6.77E-06 |
| P38 MAPK |  | Group | Activated | 4.929 | 8.91E-16 |
| Tgf beta |  | Group | Activated | 4.107 | 4.27E-11 |
| ERK |  | Group | Activated | 4.036 | 8.87E-10 |
| Jnk |  | Group | Activated | 3.898 | 2.67E-08 |
| Creb |  | Group | Activated | 3.586 | 3.99E-06 |
| ERK1/2 |  | Group | Activated | 3.524 | 3.14E-07 |
| TGM2 | 0.159 | Enzyme | Activated | 4.61 | 5.56E-10 |
| NCF1 | 1.039 | Enzyme | Activated | 3.258 | 5.08E-05 |
| IL1B | 0.926 | Cytokine | Activated | 6.457 | 2.78E-18 |
| TNF | 1.326 | Cytokine | Activated | 6.082 | 9.03E-33 |
| IL1A | 0.614 | Cytokine | Activated | 5.812 | 3E-07 |
| IL6 | 3.577 | Cytokine | Activated | 5.476 | 8.91E-13 |
| CXCL12 | 4.081 | Cytokine | Activated | 5.316 | 2.18E-08 |
| EDN1 | -0.164 | Cytokine | Activated | 5.104 | 2.24E-11 |
| IFNG | 1.8 | Cytokine | Activated | 4.796 | 4.93E-19 |
| IL17A | 0.197 | Cytokine | Activated | 4.786 | 9.91E-07 |
| IFNA2 | 0.172 | Cytokine | Activated | 4.714 | 4.83E-07 |
| IL27 | -0.042 | Cytokine | Activated | 4.051 | 0.000058 |
| IL15 | 0.746 | Cytokine | Activated | 3.743 | 1.04E-07 |
| OSM | 2.097 | Cytokine | Activated | 3.72 | 3.22E-17 |
| IL2 |  | Cytokine | Activated | 3.536 | 1.39E-09 |
| IL5 | -0.31 | Cytokine | Activated | 3.292 | 5.13E-08 |
| CSF1 | -0.378 | Cytokine | Activated | 3.249 | 6.76E-08 |
| CCL3 | 2.007 | Cytokine | Activated | 3.24 | 2.46E-06 |
| IFNB1 | 0.763 | Cytokine | Activated | 3.193 | 1.97E-05 |
| CD40LG | 0.213 | Cytokine | Activated | 3.096 | 3.37E-08 |
| TNFSF11 | 0.231 | Cytokine | Activated | 3.089 | 3.15E-09 |

**Supplementary Table S14. Downstream targets of JAK2 altered in residual CRC tissues after RT (GSE15781).**

| Target | Expr Log Ratio | Molecule Type | JAK2 |
| --- | --- | --- | --- |
| CDH1 | -4.016 | Other | Inhibited |
| MYC | -1.32 | Transcription regulator | Inhibited |
| RARA | 0.783 | Ligand-dependent nuclear receptor | Inhibited |
| RBP1 | 1.854 | Transporter | Inhibited |
| ACTB | 0.825 | Oher | Affected |
| ACTN1 | 1.863 | Transcription regulator | Affected |
| CDC25A | -0.544 | Phosphatase | Affected |
| CST7 | 1.299 | Other | Affected |
| DCXR | -0.804 | Enzyme | Affected |
| FCGR2B | 2.304 | Transmembrane receptor | Affected |
| HTR2A | 1.645 | G-protein coupled receptor | Affected |
| LAT2 | 1.309 | Other | Affected |
| LCN2 | -3.531 | Transporter | Affected |
| LMO2 | 1.786 | Transcription regulator | Affected |
| NDN | 2.35 | Transcription regulator | Affected |
| NKG7 | 0.683 | Other | Affected |
| ODC1 | -1.302 | Enzyme | Affected |
| PHLDA3 | 2.425 | Other | Affected |
| THBS1 | 2.209 | Other | Affected |
| TP53 | -0.976 | Transcription regulator | Affected |
| TRIB2 | 1.315 | Kinase | Affected |
| VLDLR | 2.157 | Transporter | Affected |
| CCL2 | 2.78 | Cytokine | Activated |
| CCL5 | 1.211 | Cytokine | Activated |
| CCND2 | 0.836 | Other | Activated |
| CD36 | 1.832 | Transmembrane receptor | Activated |
| CDKN1A | 1.733 | Kinase | Activated |
| CYBB | 1.374 | Enzyme | Activated |
| EGR1 | 1.816 | Transcription regulator | Activated |
| ESR1 | 2.246 | Ligand-dependent nuclear receptor | Activated |
| FGF2 | 3.098 | Growth factor | Activated |
| FOS | 1.578 | Transcription regulator | Activated |
| GBP2 | 1.3 | Enzyme | Activated |
| ICAM1 | 1.4 | Transmembrane receptor | Activated |
| IL6 | 3.577 | Cytokine | Activated |
| JAK2 | 0.988 | Kinase | Activated |
| OSMR | 1.951 | Transmembrane receptor | Activated |
| PTGS2 | 2.648 | Enzyme | Activated |
| STAT1 | 0.693 | Transcription regulator | Activated |
| STAT3 | 0.546 | Transcription regulator | Activated |
| TNF | 1.326 | Cytokine | Activated |

**Supplementary Figure Legends**

**Supplementary Fig. S1.**

(A) Real-time qPCR analysis of JAK2 knockdown in HCT116 cells. Based on the mRNA levels, the siRNA showing the most efficient knockdown effect was selected. (B) Real-time qPCR analysis (left) and Western blot analysis (right) of HCT116 cells transfected with JAK2-targeting shRNA (C) Real-time qPCR analysis (left) and Western blot analysis (right) of LoVo cells transfected with JAK2-targeting siRNA (D) The MTT assay was performed to assess cell viability. HCT116 cells transfected with siRNA sequence #3 were seeded in 96-well plates after being subjected to various doses of radiation. Cell viability was quantified after 72 hours of incubation. (E and F) The IC_50_ of Stattic was evaluated in HCT116 and LoVo cells by the MTT assay. (G and H) STAT family protein expression in HCT116 and LoVo cells under the conditions of radiation and Stattic treatment was confirmed by Western blot. (I and J) Clonogenic assays were performed using HCT116 cells. Cells were treated with radiation at various doses ranging from 1 to 10 Gy with or without (I) JAK2 silencing or (J) Stattic treatment. And then, they were seeded in 12-well plates and observed for 2 weeks. The surviving colonies were visualized by crystal violet staining. Bar graphs represent the mean ± SD (n=3), and statistical analysis was performed by t-test or one-way ANOVA with Dunnett’s multiple comparison; *, **, and *** indicate p<0.05, p<0.01, and p<0.001, respectively.

**Supplementary Fig. S2.**

(A and B) Immunofluorescence assays were performed to visualize the target proteins JAK2 (A) and p-STAT3 (B) in primary tumors collected from the *in vivo* xenograft model (n=9/group). (C and D) The anchorage-independent growth of cells was estimated by soft agar assays. LoVo cells with JAK2 knockdown (C) or Stattic treatment (D) were irradiated (2 Gy), seeded in agar-layered plates and incubated for 2 months. (E andF) Effects of JAK2 knockdown or Stattic treatment on the apoptotic cell population (Annexin V+) in HCT116 (E) and LoVo cells (F) at 24 hours after radiation treatment (2 Gy). (G and H) Immunofluorescence assays were performed to visualize the target proteins Ki67 (G) and TUNEL (H) in primary tumors collected from the *in vivo* xenograft model (n=9/group). Nuclei were stained with DAPI and matched with H&E stained images. Bar graphs represent the mean ± SD (n=3), and statistical analysis was performed by t-test or one-way ANOVA with Dunnett’s multiple comparison; *, **, and *** indicate p<0.05, p<0.01, and p<0.001, respectively.

**Supplementary Fig. S3.**

(A) Monolayer-cultured HCT116 cells and sphere-cultured HCT116 cells were validated by performing real-time qPCR using stem markers (*POU5F1*, *SOX2*, *NANOG*), differentiation markers (*ALPI*, *FABP1*) and JAK2. (B) Immunofluorescence assays were performed to compare the JAK2 expression between monolayer and sphere-cultured HCT116 cells. Blue indicates nuclei, and red indicates JAK2. (C) CD44v6+ cells and CD44v6- cells were sorted by FACS. (D) FACS analysis using Ki67 staining was performed to compare the proliferating cells between the CD44v6+ and CD44v6‑ populations following radiation. (E) FACS analysis using Annexin V staining was performed to compare the apoptotic cells between CD44v6+ and CD44v6- populations following radiation. (F) FACS analysis using γH2AX staining was performed to compare the radiation-induced DNA damage between the CD44v6+ and CD44v6‑ cell populations. (G) Comet assay was performed to compate the radiation-induced DNA damage accumulation between the CD44v6+ and CD44v6- populations following radiation. (H) Phospho-STAT3 expression was compared between the CD44v6+ and CD44v6‑ populations in HCT116, LoVo and patient-derived cells by FACS analysis. (I) Effects of JAK2 knockdown on mRNA levels of various CSC-related genes in HCT116 cells. (J and K) To compare the stem cell frequencies between vehicle and Stattic-treated cells, a limiting dilution assay was performed. (L) Effects of JAK2 knockdown on sphere-forming efficiency of HCT116 cells with or without radiation treatment. (M) An immunofluorescence assay was performed to visualize the target protein CD44v6 in the primary tumor collected from the *in vivo* xenograft model (n=9/group). Nuclei were stained with DAPI and matched with H&E stained images. (N-Q) The CD44v6+ population enriched by radiation was measured by FACS analysis at 24 hours after radiation with or without JAK2 silencing/Stattic treatment. Bar graphs represent the mean ± SD (n=3), and statistical analysis was performed by t-test or one-way ANOVA with Dunnett’s multiple comparison; *, **, and *** indicate p<0.05, p<0.01, and p<0.001, respectively.

**Supplementary Fig. S4.**

(A) An immunofluorescence assay was performed to visualize the target proteins CCND2 in primary tumors collected from an *in vivo* xenograft model (n=9/group). Nuclei were stained with DAPI and matched with H&E stained images. Bar graphs represent the mean ± SD (n=3), and statistical analysis was performed by one-way ANOVA with Dunnett’s multiple comparison; *, **, and *** indicate p<0.05, p<0.01, and p<0.001, respectively.

**Supplementary Fig. S5**

(A) The efficiencies of three siCCND2 sequences were evaluated by real-time qPCR analysis (left) and Western blot analysis (right). (B) The selected efficient siRNA sequence was confirmed in the LoVo cell line by real-time qPCR analysis (left) and Western blot analysis (right). (C) The MTT assay was performed to assess cell viability. HCT116 cells transfected with siRNA sequence #2 were seeded in 96-well plates after being subjected to various doses of radiation. Cell viability was quantified after 72 hours of incubation. (D) Effect of CCND2 knockdown on the apoptotic cell population (Annexin V+) in HCT116 cells at 24 hours after radiation treatment (2 Gy). (E) Sphere-formation assay was performed to estimate the CCND2 knockdown effect on sphere-forming efficiency before and after radiation treatment in HCT116 cells. (F) The radiation-induced CD44v6+ cell population was measured by FACS analysis 24 hours after radiation under CCND2 knockdown conditions. Bar graphs represent the mean ± SD (n=3), and statistical analysis was performed by one-way ANOVA with Dunnett’s multiple comparison; *, **, and *** indicate p<0.05, p<0.01, and p<0.001, respectively.

**Supplementary Fig. S6**

(A) A significantly positive correlation between JAK2 and CCND2 was observed in various cancers, including breast (GSE3494), lung (GSE19804), melanoma (GSE65904) and renal (GSE2712) cancer. (B) shCTRL- or shJAK2 vector-transfected HCT116 cells were injected into the tail veins of mice. At 28 days after the injection, metastatic nodules on the lungs were visualized and counted by India ink staining. The dots represent the number of metastatic nodules from each mouse, and the lines show the mean±SEM (n=9/group). (C) Predicted binding sites of AP-1, c-Jun and c-Fos on the JAK2 promoter region according to ALLGEN PROMO database version 3.0.2. (D) Effects of CCND2 knockdown on mRNA levels of various radioresistance genes. Bar graphs represent the mean ± SD (n=3), and statistical analysis was performed by one-way ANOVA with Dunnett’s multiple comparison; *, **, and *** indicate p<0.05, p<0.01, and p<0.001, respectively.
