# Supplementary figures and images for: The JAK2/STAT3/CCND2 Axis promotes colorectal Cancer stem cell persistence and radioresistance
Source: J Exp Clin Cancer Res. 2019 Sep 11;38:399. doi: 10.1186/s13046-019-1405-7 (PMC6737692; doi:10.1186/s13046-019-1405-7)

Supplementary Figure S1

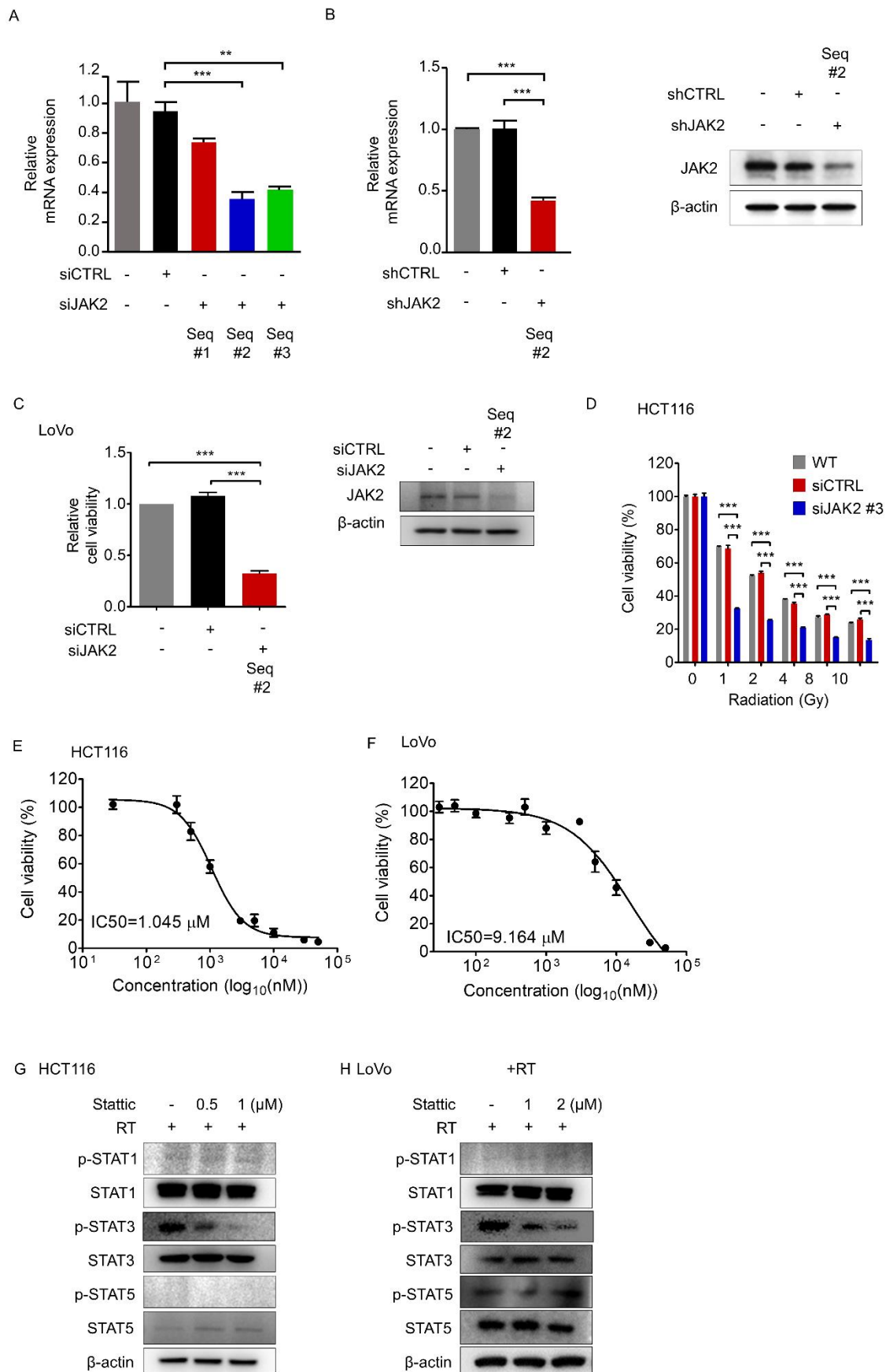

Supplementary Figure S1

I

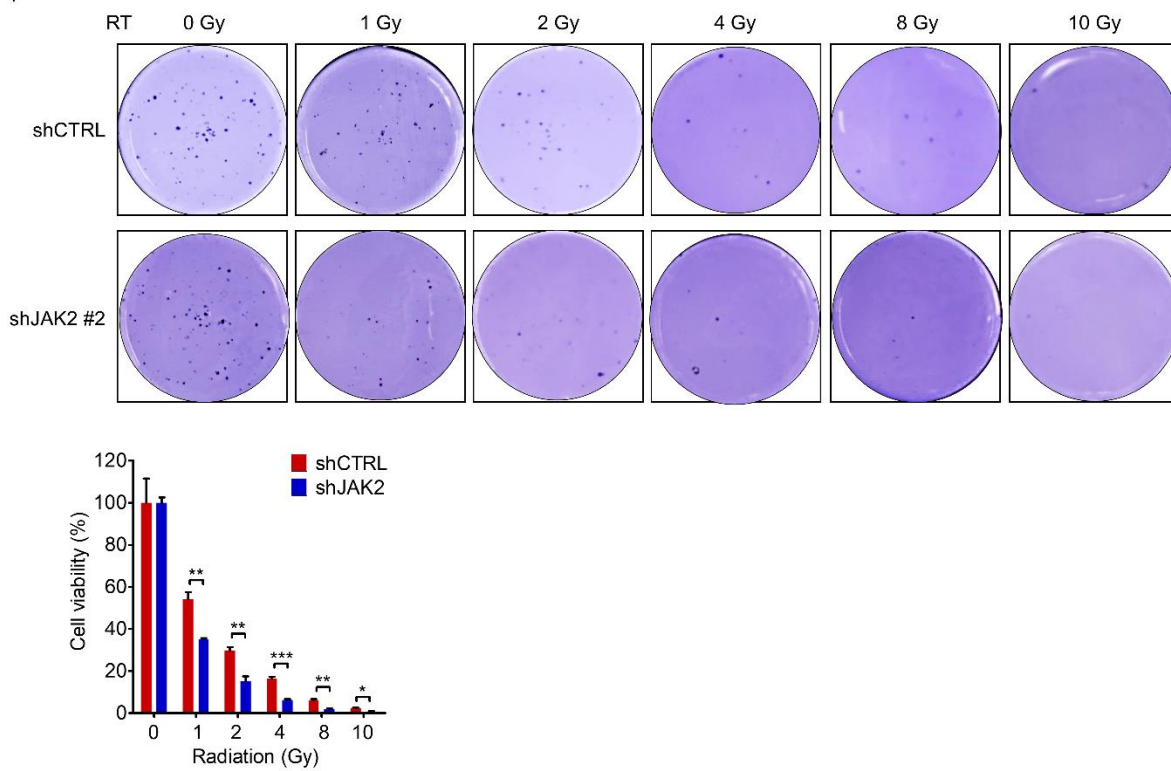

J

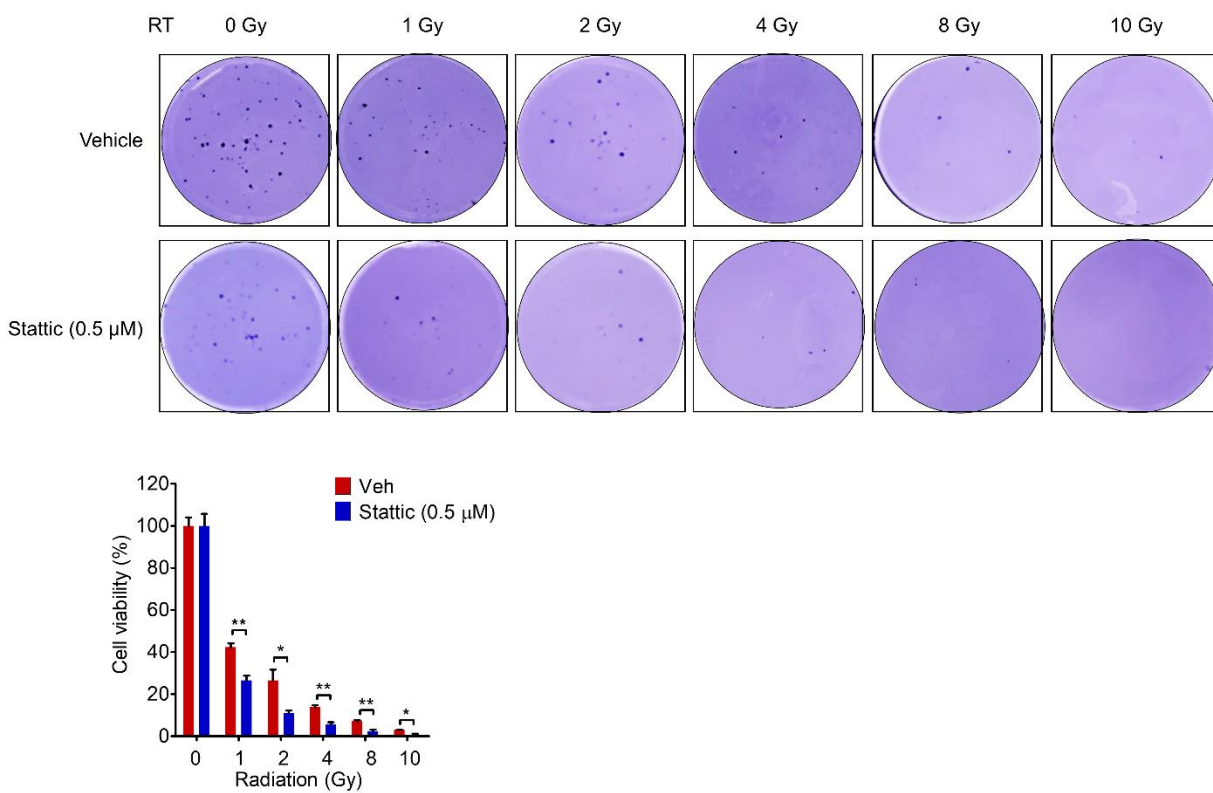

Supplement: Supplementary file 2 — Figure S1. (A) Real-time qPCR analysis of JAK2 knockdown in HCT116 cells. Based on the mRNA levels, the siRNA showing the most efficient knockdown effect was selected. (B) Real-time qPCR analysis (left) and Western blot analysis (right) of HCT116 cells transfected with JAK2-targeting shRNA (C) Real-time qPCR analysis (left) and Western blot analysis (right) of LoVo cells transfected with JAK2-targeting siRNA (D) The MTT assay was performed to assess cell viability. HCT116 cells transfected with siRNA sequence #3 were seeded in 96-well plates after being subjected to various doses of radiation. Cell viability was quantified after 72 hours of incubation. (E and F) The IC50 of Stattic was evaluated in HCT116 and LoVo cells by the MTT assay. (G and H) STAT family protein expression in HCT116 and LoVo cells under the conditions of radiation and Stattic treatment was confirmed by Western blot. (I and J) Clonogenic assays were performed using HCT116 cells. Cells were treated with radiation at various doses ranging from 1 to 10 Gy with or without (I) JAK2 silencing or (J) Stattic treatment. And then, they were seeded in 12-well plates and observed for 2 weeks. The surviving colonies were visualized by crystal violet staining. Bar graphs represent the mean ± SD (n = 3), and statistical analysis was performed by t-test or one-way ANOVA with Dunnett’s multiple comparison; *, **, and *** indicate p < 0.05, p < 0.01, and p < 0.001, respectively. (PDF 463 kb) [file 13046_2019_1405_MOESM2_ESM.pdf]

Supplementary Figure S2.

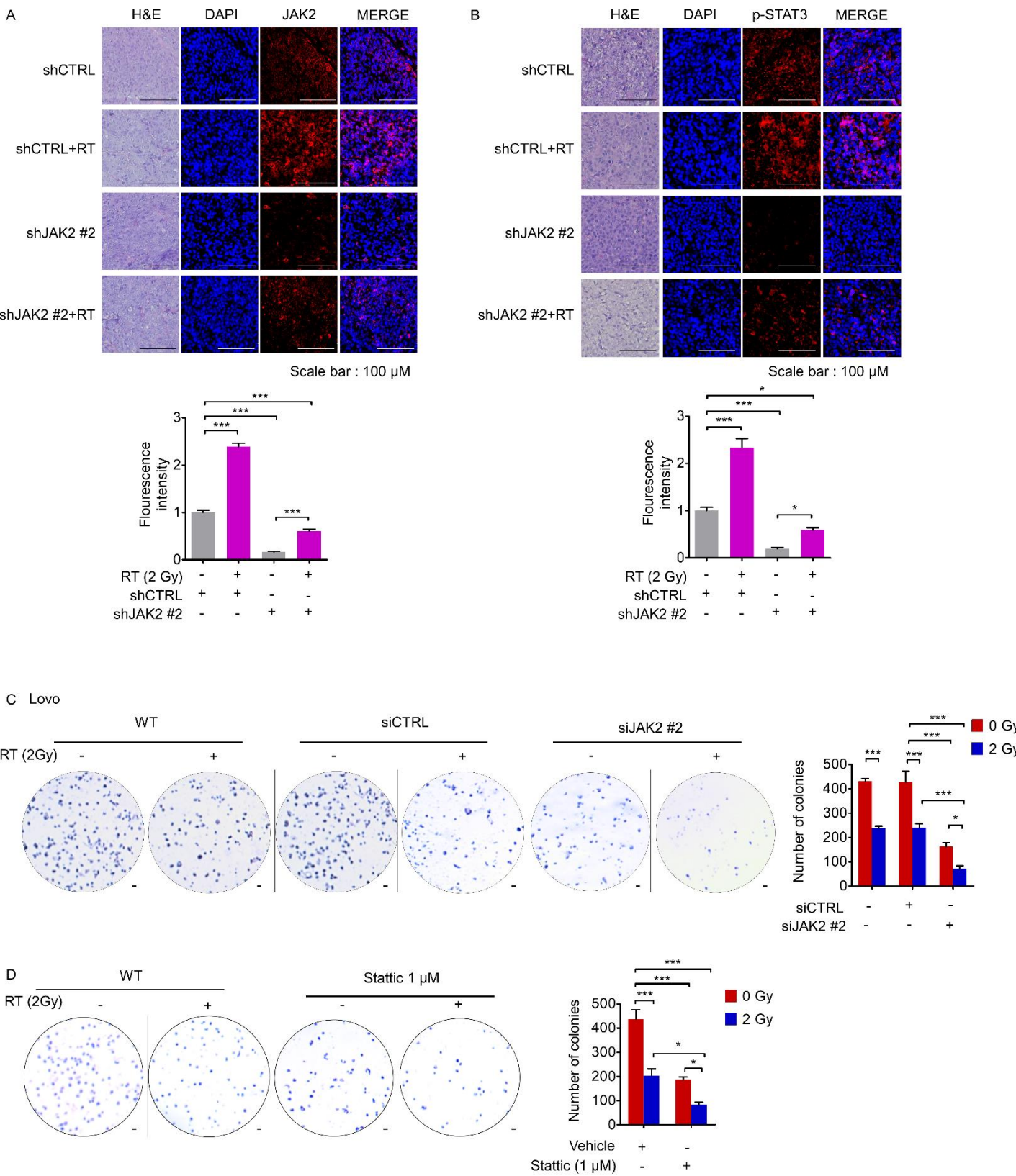

Supplementary Figure S2.

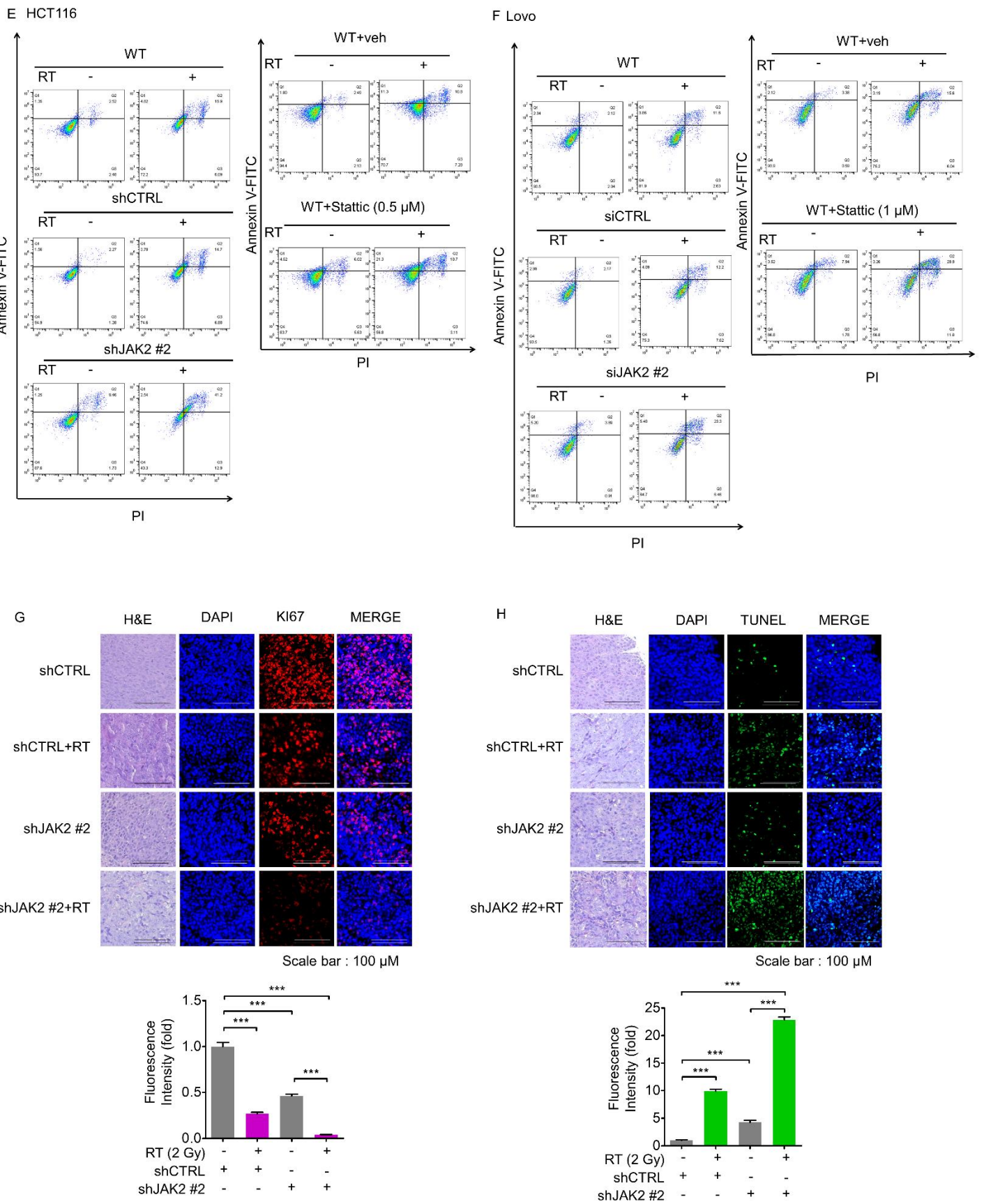

Supplement: Supplementary file 3 — Figure S2. (A and B) Immunofluorescence assays were performed to visualize the target proteins JAK2 (A) and p-STAT3 (B) in primary tumors collected from the in vivo xenograft model (n = 9/group). (C and D) The anchorage-independent growth of cells was estimated by soft agar assays. LoVo cells with JAK2 knockdown (C) or Stattic treatment (D) were irradiated (2 Gy), seeded in agar-layered plates and incubated for 2 months. (E andF) Effects of JAK2 knockdown or Stattic treatment on the apoptotic cell population (Annexin V+) in HCT116 (E) and LoVo cells (F) at 24 hours after radiation treatment (2 Gy). (G and H) Immunofluorescence assays were performed to visualize the target proteins Ki67 (G) and TUNEL (H) in primary tumors collected from the in vivo xenograft model (n = 9/group). Nuclei were stained with DAPI and matched with H&E stained images. Bar graphs represent the mean ± SD (n = 3), and statistical analysis was performed by t-test or one-way ANOVA with Dunnett’s multiple comparison; *, **, and *** indicate p < 0.05, p < 0.01, and p < 0.001, respectively. (PDF 738 kb) [file 13046_2019_1405_MOESM3_ESM.pdf]

Supplementary Figure S3

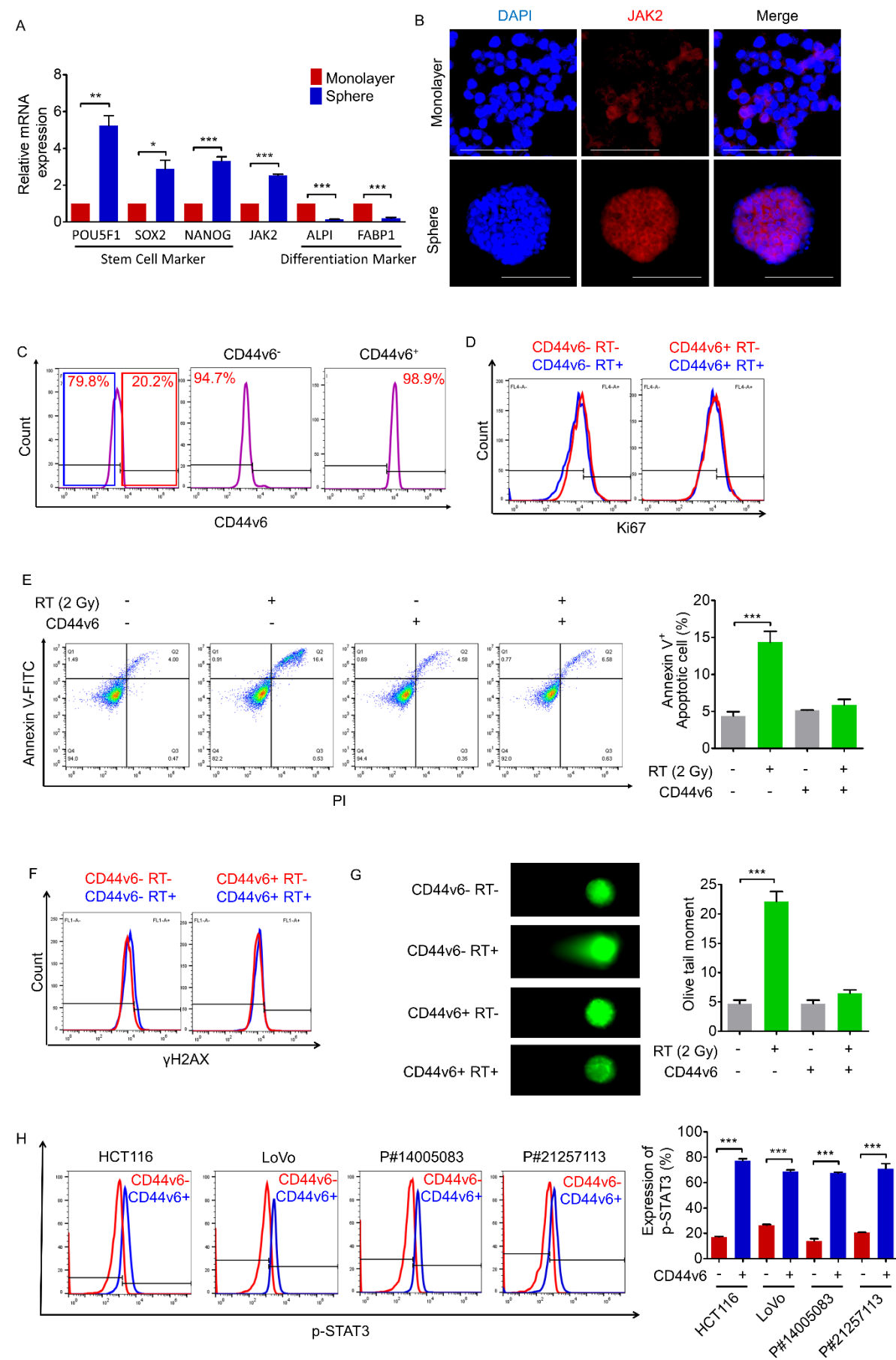

Supplementary Figure S3

I

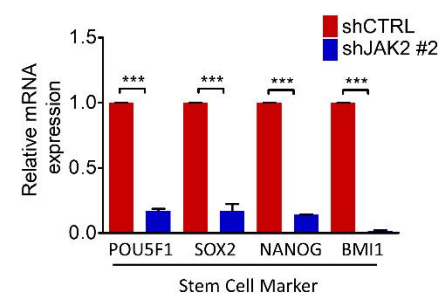

J

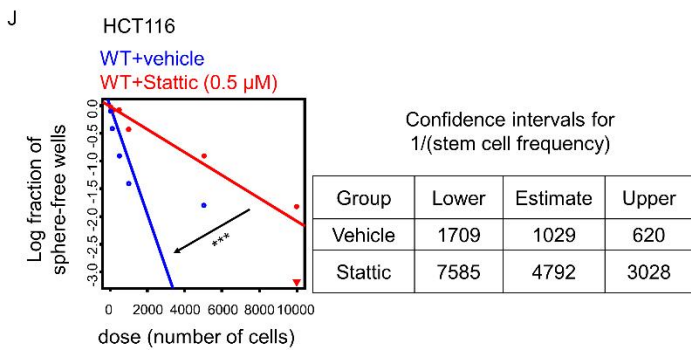

K

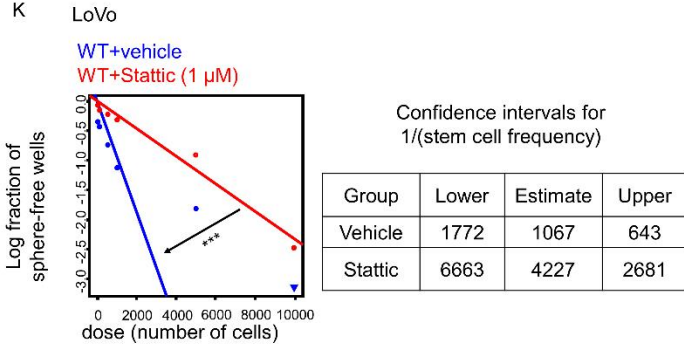

L

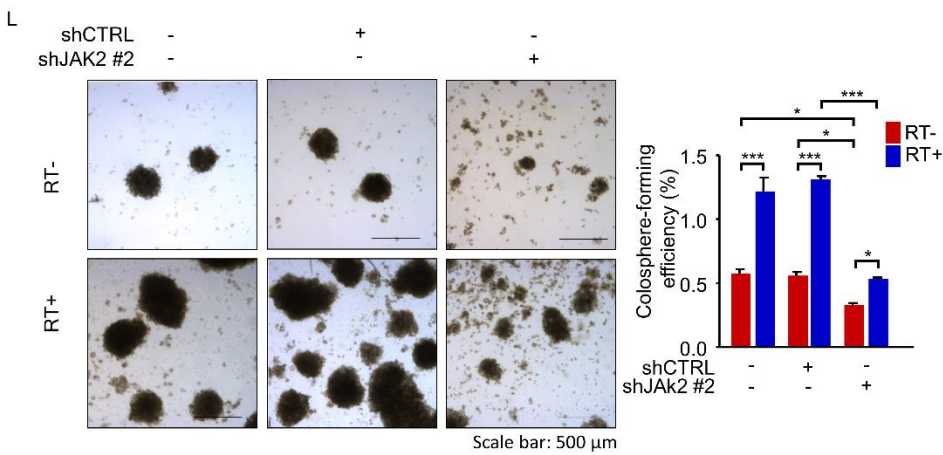

M

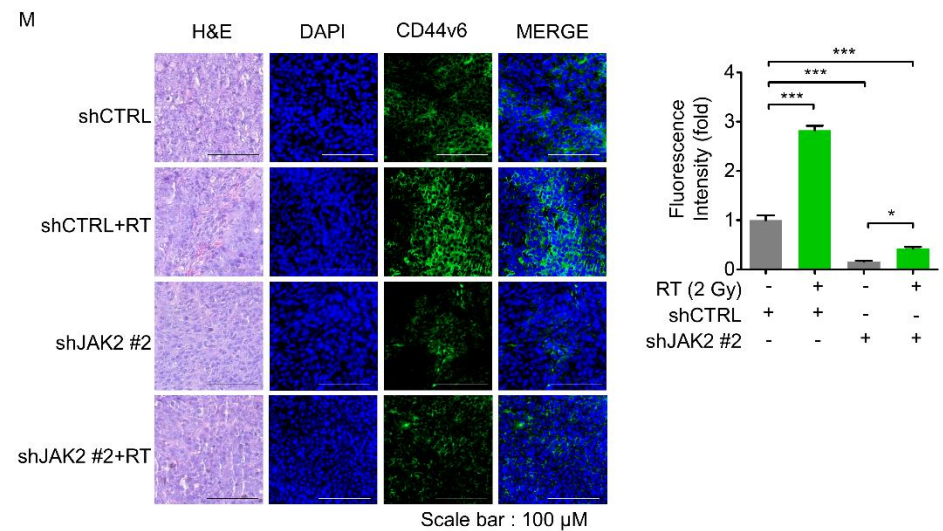

Supplementary Figure S3

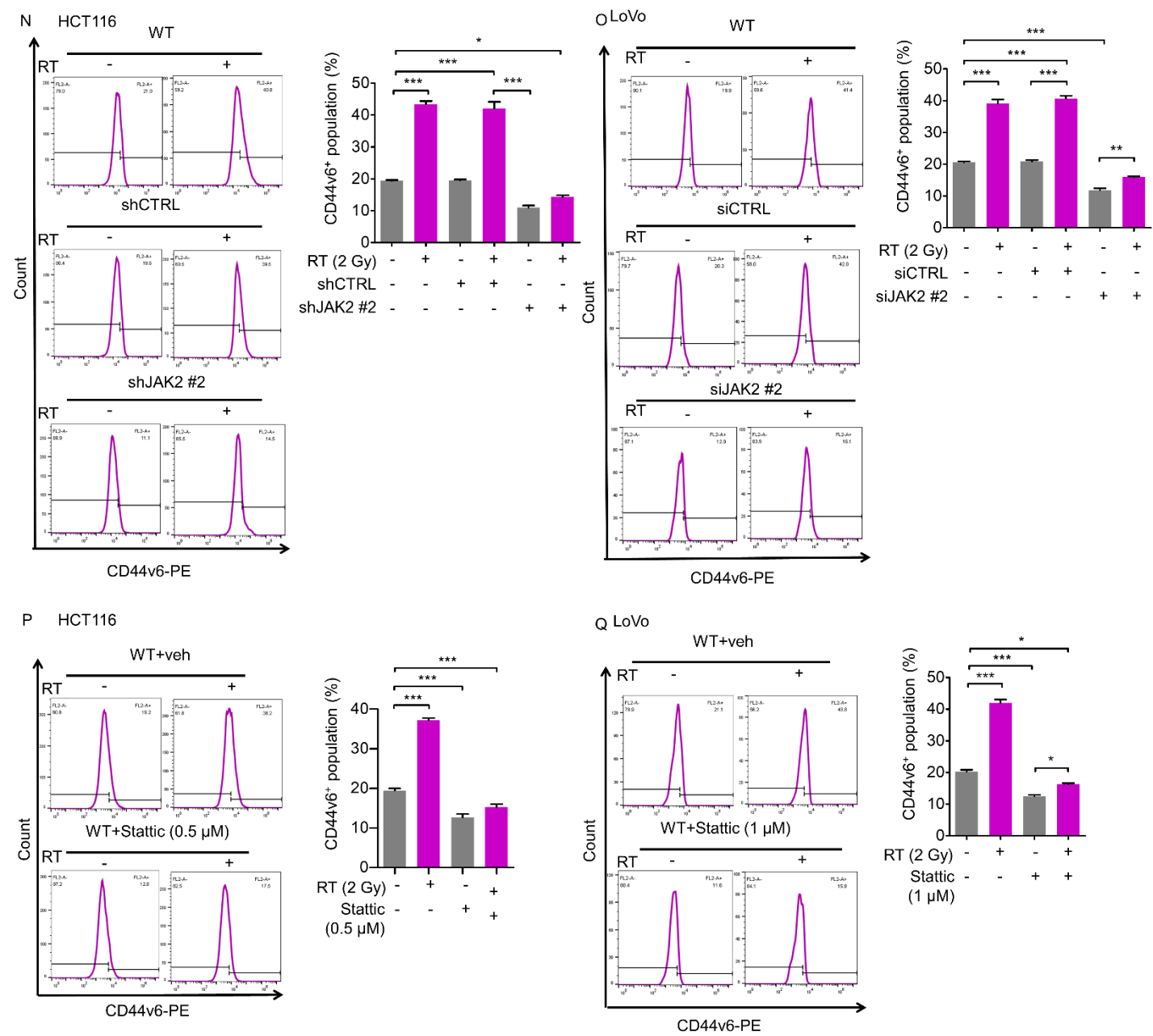

Supplement: Supplementary file 4 — Figure S3. (A) Monolayer-cultured HCT116 cells and sphere-cultured HCT116 cells were validated by performing real-time qPCR using stem markers (POU5F1, SOX2, NANOG), differentiation markers (ALPI, FABP1) and JAK2. (B) Immunofluorescence assays were performed to compare the JAK2 expression between monolayer and sphere-cultured HCT116 cells. Blue indicates nuclei, and red indicates JAK2. (C) CD44v6+ cells and CD44v6- cells were sorted by FACS. (D) FACS analysis using Ki67 staining was performed to compare the proliferating cells between the CD44v6+ and CD44v6- populations following radiation. (E) FACS analysis using Annexin V staining was performed to compare the apoptotic cells between CD44v6+ and CD44v6- populations following radiation. (F) FACS analysis using γH2AX staining was performed to compare the radiation-induced DNA damage between the CD44v6+ and CD44v6- cell populations. (G) Comet assay was performed to compate the radiation-induced DNA damage accumulation between the CD44v6+ and CD44v6- populations following radiation. (H) Phospho-STAT3 expression was compared between the CD44v6+ and CD44v6- populations in HCT116, LoVo and patient-derived cells by FACS analysis. (I) Effects of JAK2 knockdown on mRNA levels of various CSC-related genes in HCT116 cells. (J and K) To compare the stem cell frequencies between vehicle and Stattic-treated cells, a limiting dilution assay was performed. (L) Effects of JAK2 knockdown on sphere-forming efficiency of HCT116 cells with or without radiation treatment. (M) An immunofluorescence assay was performed to visualize the target protein CD44v6 in the primary tumor collected from the in vivo xenograft model (n = 9/group). Nuclei were stained with DAPI and matched with H&E stained images. (N-Q) The CD44v6+ population enriched by radiation was measured by FACS analysis at 24 h after radiation with or without JAK2 silencing/Stattic treatment. Bar graphs represent the mean ± SD (n = 3), and statistical analysis was performed by t- [file 13046_2019_1405_MOESM4_ESM.pdf]

Supplementary Figure S4

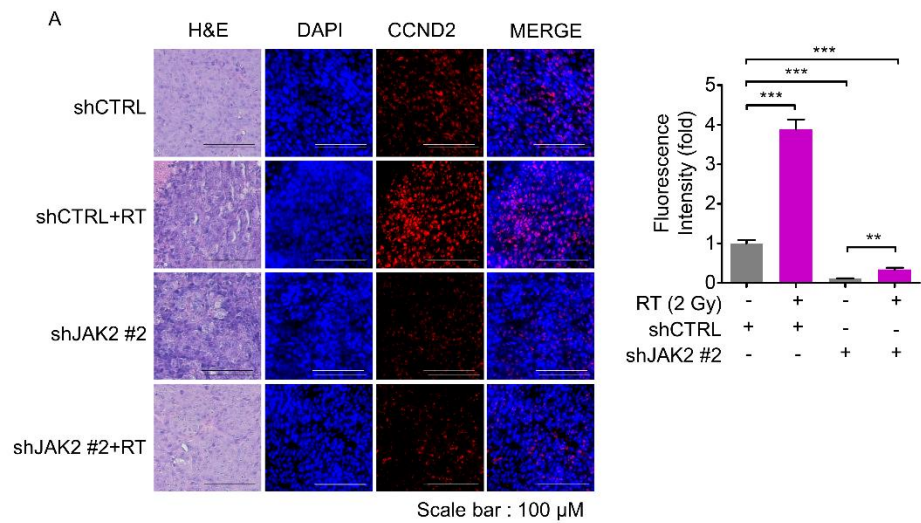

Supplement: Supplementary file 5 — Figure S4. (A) An immunofluorescence assay was performed to visualize the target proteins CCND2 in primary tumors collected from an in vivo xenograft model (n = 9/group). Nuclei were stained with DAPI and matched with H&E stained images. Bar graphs represent the mean ± SD (n = 3), and statistical analysis was performed by one-way ANOVA with Dunnett’s multiple comparison; *, **, and *** indicate p < 0.05, p < 0.01, and p < 0.001, respectively. (PDF 151 kb) [file 13046_2019_1405_MOESM5_ESM.pdf]

Supplementary Figure S5

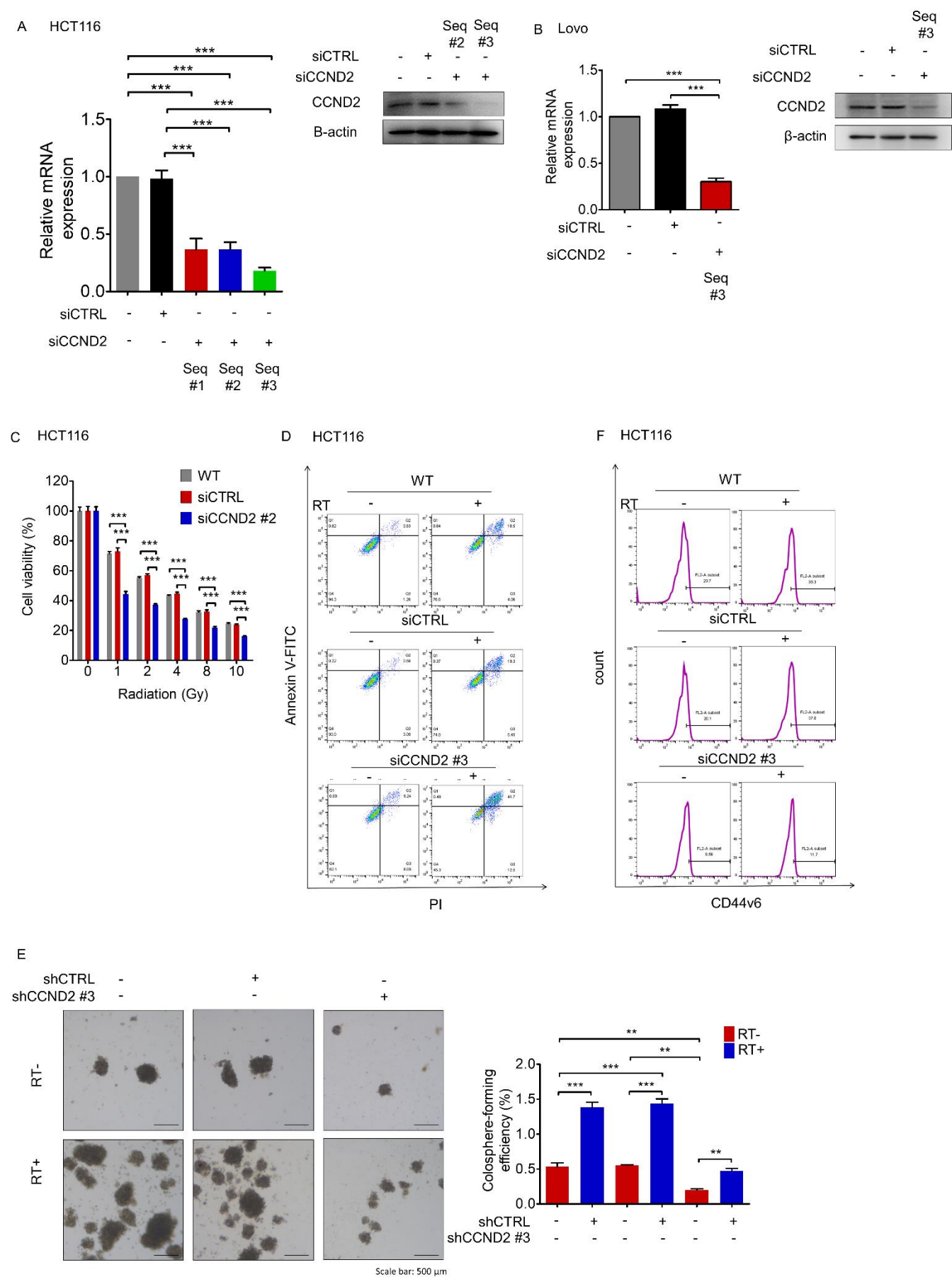

Supplement: Supplementary file 6 — Figure S5. (A) The efficiencies of three siCCND2 sequences were evaluated by real-time qPCR analysis (left) and Western blot analysis (right). (B) The selected efficient siRNA sequence was confirmed in the LoVo cell line by real-time qPCR analysis (left) and Western blot analysis (right). (C) The MTT assay was performed to assess cell viability. HCT116 cells transfected with siRNA sequence #2 were seeded in 96-well plates after being subjected to various doses of radiation. Cell viability was quantified after 72 h of incubation. (D) Effect of CCND2 knockdown on the apoptotic cell population (Annexin V+) in HCT116 cells at 24 h after radiation treatment (2 Gy). (E) Sphere-formation assay was performed to estimate the CCND2 knockdown effect on sphere-forming efficiency before and after radiation treatment in HCT116 cells. (F) The radiation-induced CD44v6+ cell population was measured by FACS analysis 24 h after radiation under CCND2 knockdown conditions. Bar graphs represent the mean ± SD (n = 3), and statistical analysis was performed by one-way ANOVA with Dunnett’s multiple comparison; *, **, and *** indicate p < 0.05, p < 0.01, and p <0.001, respectively. (PDF 265 kb) [file 13046_2019_1405_MOESM6_ESM.pdf]

Supplementary Figure S6

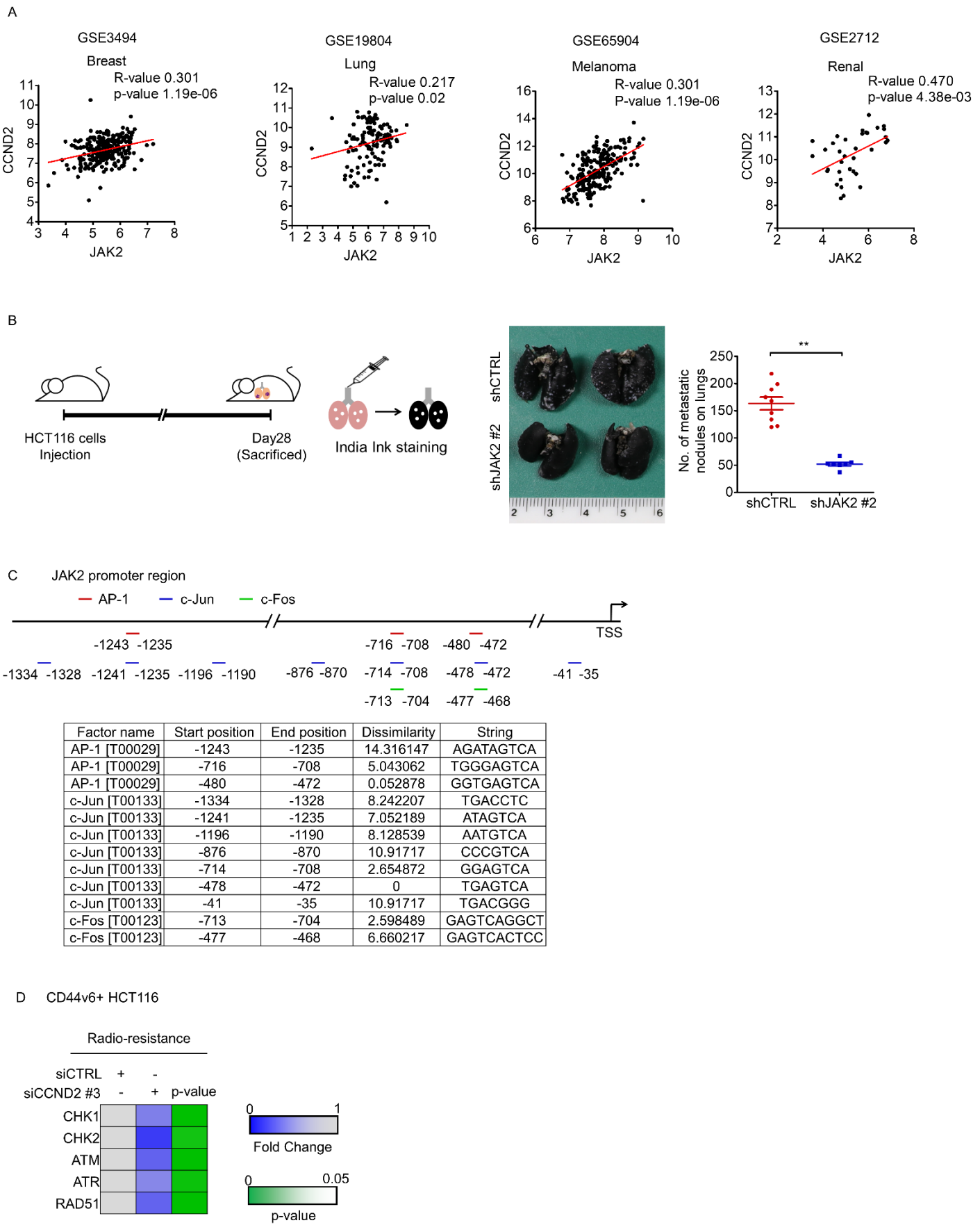

Supplement: Supplementary file 7 — Figure S6. (A) A significantly positive correlation between JAK2 and CCND2 was observed in various cancers, including breast (GSE3494), lung (GSE19804), melanoma (GSE65904) and renal (GSE2712) cancer. (B) shCTRL- or shJAK2 vector-transfected HCT116 cells were injected into the tail veins of mice. At 28 days after the injection, metastatic nodules on the lungs were visualized and counted by India ink staining. The dots represent the number of metastatic nodules from each mouse, and the lines show the mean ± SEM (n = 9/group). (C) Predicted binding sites of AP-1, c-Jun and c-Fos on the JAK2 promoter region according to ALLGEN PROMO database version 3.0.2. (D) Effects of CCND2 knockdown on mRNA levels of various radioresistance genes. Bar graphs represent the mean ± SD (n = 3), and statistical analysis was performed by one-way ANOVA with Dunnett’s multiple comparison; *, **, and *** indicate p < 0.05, p < 0.01, and p < 0.001, respectively. (PDF 410 kb) [file 13046_2019_1405_MOESM7_ESM.pdf]
